# From Low- to High-Entropy PBAs and Their Derivatives for Lithium–Sulfur Batteries

**DOI:** 10.1021/acsomega.5c04943

**Published:** 2025-08-22

**Authors:** Josué M. Gonçalves, Érick A. Santos, Murilo M. Amaral, Edson Nossol, Rafael O. Figueiredo, Hudson Zanin

**Affiliations:** † Mackenzie Institute for Research in Graphene and Nanotechnologies (MackGraphe), 42524 Mackenzie Presbyterian Institute, Consolação Street 930, São Paulo, São Paulo 01302-907, Brazil; ‡ Advanced Energy Storage Division, Carbon Sci-Tech Laboratories; Center for Innovation on New Energies; School of Electrical and Computer Engineering, University of Campinas, Av Albert Einstein 400, Campinas, São Paulo 13083-852, Brazil; § Brazilian Center for Research in Energy and Materials (CNPEM) − Brazilian Synchrotron Light Laboratory (LNLS), Campinas, São Paulo 13083-970, Brazil; ∥ Institute of Chemistry, 28119 Federal University of Uberlândia, Uberlândia, Minas Gerais 38400-902, Brazil; ⊥ Mackenzie School of Engineering, Mackenzie Presbyterian University, Consolação Street 930, São Paulo, São Paulo 01302-907, Brazil

## Abstract

The accelerating global demand for energy has catalyzed the pursuit of advanced, sustainable energy storage systems. Among them, lithium–sulfur (Li–S) batteries stand out for their high theoretical energy density and the abundance and low cost of sulfur. However, their practical deployment remains restricted by issues such as polysulfide dissolution, sluggish redox kinetics, and the notorious shuttle effect. Recent efforts have focused on engineering sulfur host materials and electrocatalysts to overcome these limitations, particularly through the use of polar inorganic compounds that interact strongly with lithium polysulfides (LiPSs) to improve conversion efficiency and cycle stability. In this context, new materials with configurational entropy ranging from low to high entropy have emerged as a new class of functional materials, offering unprecedented structural and compositional tunability. Among them, Prussian blue analogues (PBAs) have gained increasing attention due to their open frameworks, controllable composition, and redox-active sites. Moreover, PBAs represent highly versatile precursors for the rational design and synthesis of advanced catalytic materials, encompassing alloys, metal oxides, metal sulfides, and metal phosphides, among other functional compounds. This review provides a comprehensive and critical assessment of the progress in the application of low-, medium-, and high-entropy PBAs and their derivatives as solid catalysts in Li–S batteries. We clarify the definitions of configurational entropy in PBAs and highlight the current misuse of the term in the literature. The review also addresses synthetic strategies for multielement PBAs, evaluates their physicochemical and catalytic properties, and correlates these features with electrochemical performance. Finally, we identify emerging design trends, key challenges, and future perspectives for the rational development of entropy-tailored PBAs in next-generation Li–S batteries.

## Graphical abstract

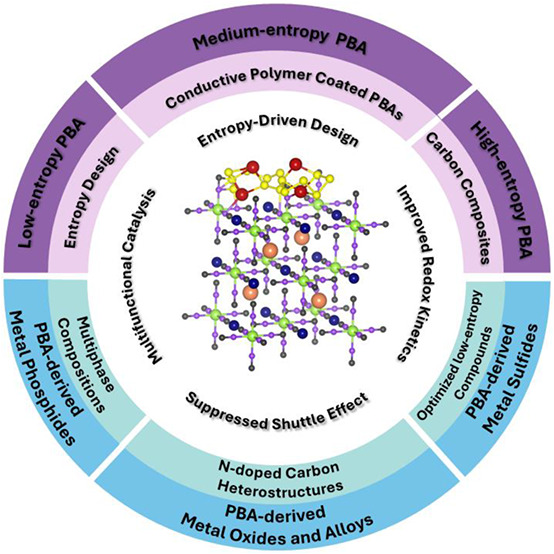

## 1 Introduction

The swift evolution of society and the global upsurge in energy consumption underscore the imperative for strategic research in renewable and sustainable energy conversion and storage technologies. This pursuit is essential to mitigate the challenges posed by climate change and the depletion of fossil fuels.^1,2^ In this context, fostering the advancement of energy technologies immune to weather fluctuations, unpredictable environmental changes, and intermittent characteristics stands out as a promising strategy within the energy transition framework.^3^ To this end, one of the key approaches to energy transition involves pursuing even more efficient energy storage devices.

Among the cutting-edge energy storage devices, the lithium–sulfur (Li–S) battery stands out as a promising candidate for next-generation batteries. This distinction arises from its remarkably high theoretical energy density (approximately 2600 Wh kg^–1^) and the added advantages of a cost-effective and environmentally friendly sulfur cathode material.^4,5^ Nevertheless, the commercialization of the Li–S battery faces several challenges. Its widespread adoption has been impeded by issues such as the dissolution and migration of lithium polysulfides (LiPSs) coupled with sluggish redox kinetics.^6,7^ Hence, the back-and-forth migration of insoluble LiPSs between the electrodes induces a pronounced *shuttle effect*, leading to irreversible loss of cathode active materials, particularly evident at high sulfur loading.^8^

Fortunately, recent research has put forth several strategies to tackle the mentioned challenges, resulting in notable achievements. Advancements have been made in optimizing and designing new sulfur host materials. Notably, the introduction of solid catalysts or redox mediators, uniformly dissolved in the electrolyte solutions, has shown promise in enhancing Li–S batteries.^9^ As expected, such materials can expedite electrochemical reaction kinetics and effectively mitigate the shuttle of LiPSs, which can occur through swifter electron–hole transportation processes or by overcoming excessive activation energy through the formation of a more stable transition state.^8^

Interestingly, a diversity of polar hosts based on nanostructured inorganic compounds has shown that the catalytic effect in the Li–S system is promising in solving the shuttling problem. Within this category of materials, metal particles (including alloys), metal oxides, metal sulfides, metal phosphides, and metal carbides exhibit a strong chemical affinity with LiPSs, effectively impeding their diffusion. In fact, electrodes incorporating polar hosts typically exhibit enhanced electrochemical activity compared to conventional carbon-based electrodes, which have been extensively studied. This is attributed to the nonpolar surface of carbon materials, resulting in their relatively low affinity for polar LiPSs and an inability to hinder LiPSs from shuttling effectively.^4^

Recently, the distinctive design concepts and the unexpected properties of medium-entropy materials (MEMs) and high-entropy materials (HEMs) are anticipated to usher in novel advancements in various energy conversion and storage technologies.^8^ Indeed, this emerging class of materials represents a departure from the conventional design principles centered around low-entropy materials (LEMs).^10^ As per the compositional definition, HEMs consist of at least five principal elements, each in close to equiatomic quantities, while MEMs are generally limited to three or four major element concentrations.^8,10^ Typically, compounds with one or two elements are described as LEMs. These high-entropy compounds span various material classes, including alloys, metal oxides, and metal sulfides, among others. This diversity in compositions and structures provides extensive possibilities for material design, offering a versatile platform for a wide range of applications. In recent developments, the repertoire of HEMs has been broadened to include coordination compounds, specifically in metal–organic frameworks (MOFs) and Prussian blue analogues (PBAs). This extension introduces the concepts of medium- and high-entropy into these frameworks, potentially unlocking novel opportunities for targeted applications. In fact, high-entropy coordination compounds (HE-CCs), such as high-entropy PBAs (HE-PBAs), are emerging as a promising family for the next generation of electrode materials in energy technologies.^11^ In general, PBAs have attracted significant attention due to their wide range of applications, including energy storage (e.g., supercapacitors, metal-ion batteries, and Li–S batteries), catalysis (e.g., oxygen evolution reaction and CO_2_ reduction), electrochemical sensing, water purification, and electromagnetic wave absorption.^12–14^ Notably, the remarkable characteristics of PBAs, including chemical and thermal stability, eco-friendly synthesis process, ease of tuning active metal sites, outstanding redox performance, and rapid ion insertion and extraction, stem from their open-channel architecture. Binary PBAs demonstrate greater electrochemical performance than their unary counterparts due to the enhanced redox properties arising from the interaction between the two metal ions, enhancing their polarity, which makes them promising candidates as sulfur hosts for Li–S batteries.^15^ PBAs, known for their consistent porosity and well-defined crystal architecture, have garnered attention in the field of energy storage. Their Fe^2+^/Fe^3+^ network, linked through cyanide ligands (−C≡N−), generates open frameworks that promote ion intercalation, leading to sustained capacity and enhanced cycle stability. Highly porous materials, especially those exhibiting a porous structural morphology, are essential for enabling efficient Li-ion transport and enhancing polysulfide adsorption, thereby reducing sulfur dissolution and minimizing irreversible capacity loss during cycling.^16^ Moreover, PBAs can act as a polysulfide trap to suppress the shuttle effect and function as a catalyst to accelerate polysulfide conversion, thereby enhancing their exceptional performance.^17^

It is noteworthy that our group recently provided a comprehensive summary of the advancements in utilizing MEMs and HEMs as catalysts for Li–S batteries. However, since HEMs do not consistently exhibit superior electrochemical activity compared to their lower entropy counterparts, a critical analysis of the factors influencing the performance of these materials becomes fundamentally important. Within this context and recognizing the absence of a critical review on the impact of increasing entropy in PBAs as opposed to their simpler counterparts, this review delves into the utilization of PBAs spanning from low- to high-entropy design as well as their derivatives. In fact, the design of PBA-derived materials has driven promising progress in catalytic systems for several emerging energy technologies, including Li–S batteries.

In addition, a few review articles have summarized the application of MOF materials for Li–S batteries.^18,19^ However, as far as our knowledge extends, there is currently no comprehensive review encompassing the promising results and emerging trends across PBAs at various degrees of configurational entropy and their derivatives as solid catalysts for L–S batteries. In this context, this review is dedicated to comprehensively covering the most recent advancements and offering a deeper understanding of low-entropy (LE-PBAs), medium-entropy (ME-PBAs), and HE-PBAs and their derivatives concerning their application in Li–S batteries. This contribution primarily focuses on elucidating the strategies and current trends in designing PBAs and derived materials, aiming to improve the electrochemical performance and stability of Li–S batteries (Scheme 1). The review starts by clearly defining LE-, ME-, and HEMs, emphasizing their use as catalysts in energy storage. It also provides a critical perspective on recent uses of the term “entropy”, highlighting the lack of consideration for the distinct coordination sites in PBAs and/or their compositional complexity. In addition, the main synthesis protocols and challenges of designing multielement PBAs and derivatives are highlighted. Moreover, the strategies employed to enhance electrochemical properties and catalytic activity are intricately linked to the configurational entropy of the material, as well as its composition and structure. Ultimately, this review critically discusses the merits and drawbacks of employing diverse PBA-based catalysts, providing insights into the prospects and outlining future directions in the field.

**Scheme 1:**
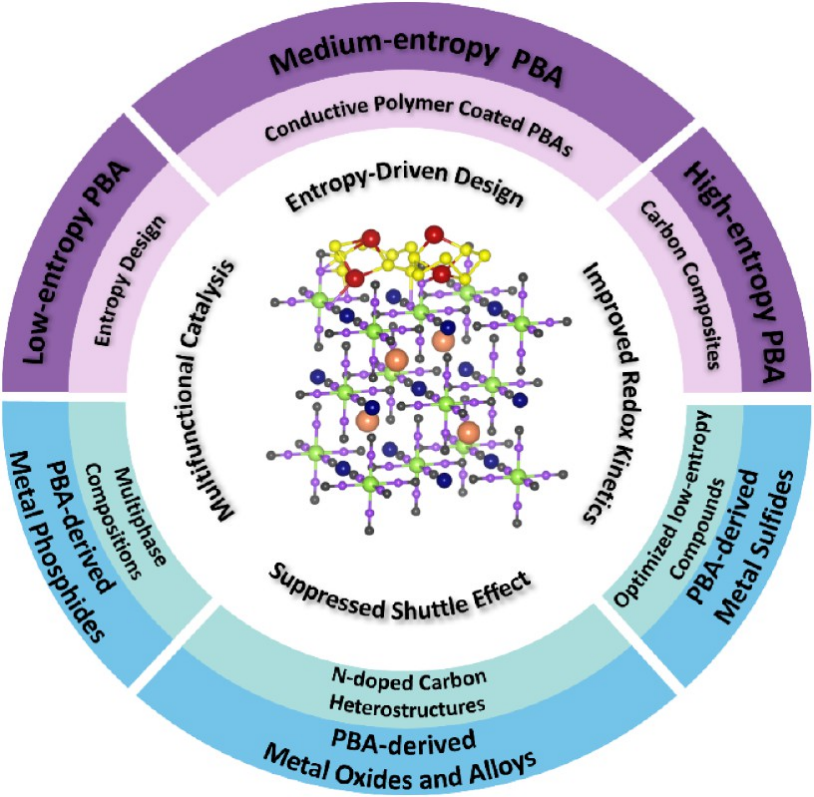
Overview of Current Trends and Strategic Approaches in the Design of PBA-Based Catalytic Materials—from Low- to High-Entropy Compositions—and Their Derivatives for Application in Li–S Batteries

## 2 Fundamentals and Challenges in Li–S Batteries

Lithium–sulfur (Li–S) batteries are attractive energy storage devices, mostly because of their high theoretical energy density and high capacity, as well as the low cost and abundance of sulfur.^20,21^ In fact, over the past decades, remarkable progress has been made in the development of Li–S batteries. Key milestones are highlighted in Figure 1, including the initial concept of sulfur cathodes, the introduction of DOL-based electrolytes, and the application of LiNO_3_ as an electrolyte additive to mitigate the shuttle effect. The encapsulation of sulfur in porous carbon marked a significant advancement in improving sulfur utilization, resulting in thousands of studies after the publication of work conducted by Dr. Linda Nazar’s group.^22^ More recently, Prussian blue (PB) and Prussian blue analogues (PBAs) have emerged as promising sulfur host materials, due to their open frameworks and redox properties. The design of high-entropy oxides and high-entropy PBAs has further enhanced electrochemical performance by promoting LiPS confinement. Furthermore, the adsorption of LiPSs by PBA-based sulfur hosts was recently confirmed via *operando* Raman spectroscopy, revealing real-time sulfur redox transitions.^16^

**Figure 1:**
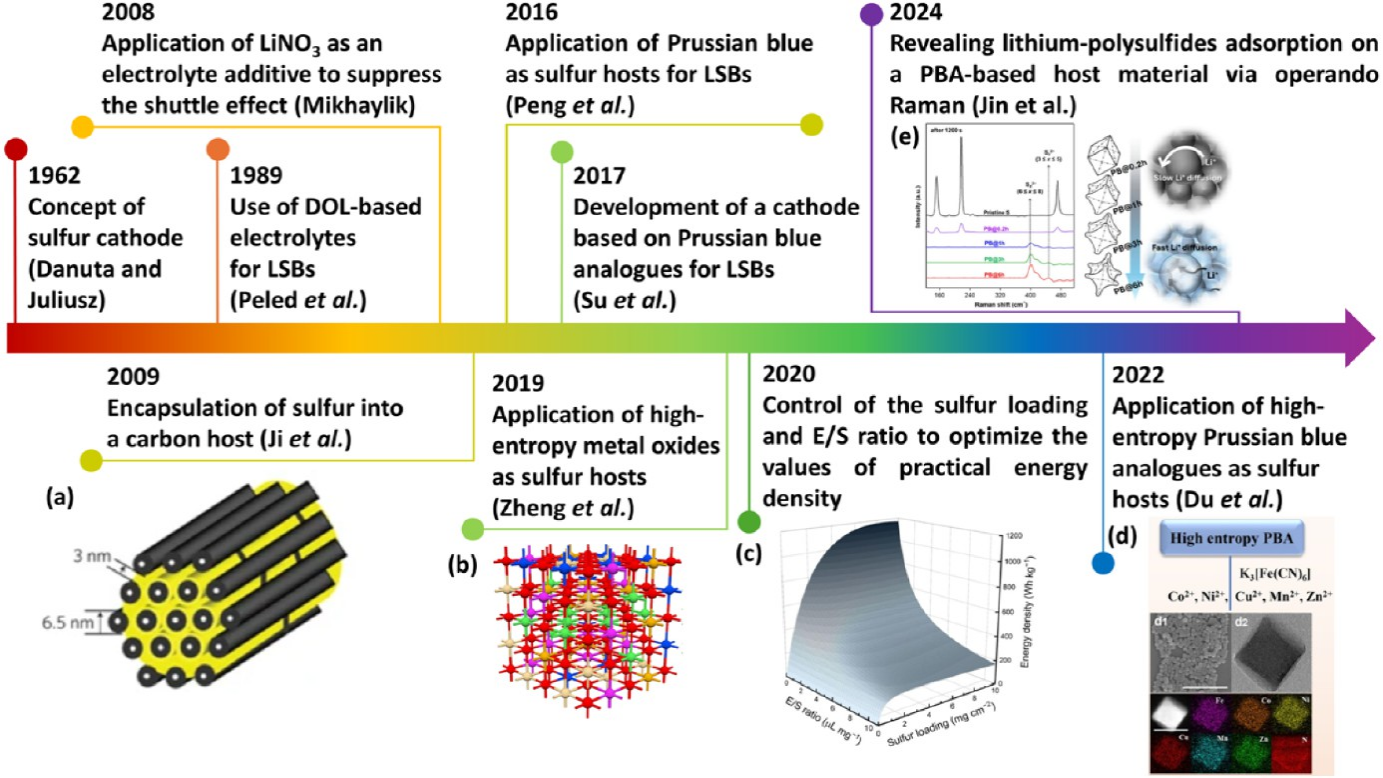
Development history of some important events in the Li–S battery (LSB) field. Timeline summarizing the designing strategies to optimize the performance of Li–S batteries, including historical remarks and the application of Prussian blue and Prussian blue analogues. References used for the timeline: Concept of a sulfur cathode,^23^ use of DOL-based electrolytes for LSBs,^24^ application of LiNO_3_ as an electrolyte additive to suppress the shuttle effect,^25^ encapsulation of sulfur into a carbon host,^22^ application of Prussian blue as sulfur hosts for LSBs,^26^ development of a cathode based on Prussian blue analogues for LSBs,^27^ application of high-entropy metal oxides as sulfur hosts,^28^ interest in LSBs with high sulfur loading and low E/S ratio to achieve high practical values of energy density,^29^ application of high-entropy Prussian blue analogues as sulfur hosts,^17^ Revealing lithium-polysulfides adsorption on a PBA-based host material via *operando* Raman.^16^ Panel (a) reprinted from ref. 22. Copyright 2009, Springer Nature Limited. Panel (b) reprinted from ref. 28. Copyright 2019, Elsevier B.V. All rights reserved. Panel (c) reprinted from ref. 29. Copyright 2020, American Chemical Society. Panel (d) reprinted from ref. 17. Copyright 2022 Wiley-VCH GmbH. Panel (e) reprinted from ref 16. Copyright 2024, American Chemical Society.

However, Li–S batteries present some challenges to overcome, including the *shuttle effect*, which basically consists of the mobility of soluble LiPSs to the lithium anode.^30^ During the polysulfide shuttle process, some soluble LiPSs might migrate to the lithium (Li) metal anode, which can be further reduced to insoluble lithium sulfide (Li_2_S) and lithium disulfide (Li_2_S_2_), culminating in the passivation of these species on the electrode surface (Figure 2a). Then, as insoluble short-chain LiPSs present slow redox kinetics, they are not completely oxidized during the charging process, resulting in a sulfur loss and decreasing the battery capacity,^31–33^ as displayed in Figure 2b. Beyond this point, sulfur has an insulating nature and presents a high volume expansion during the conversion reactions.^34^

**Figure 2:**
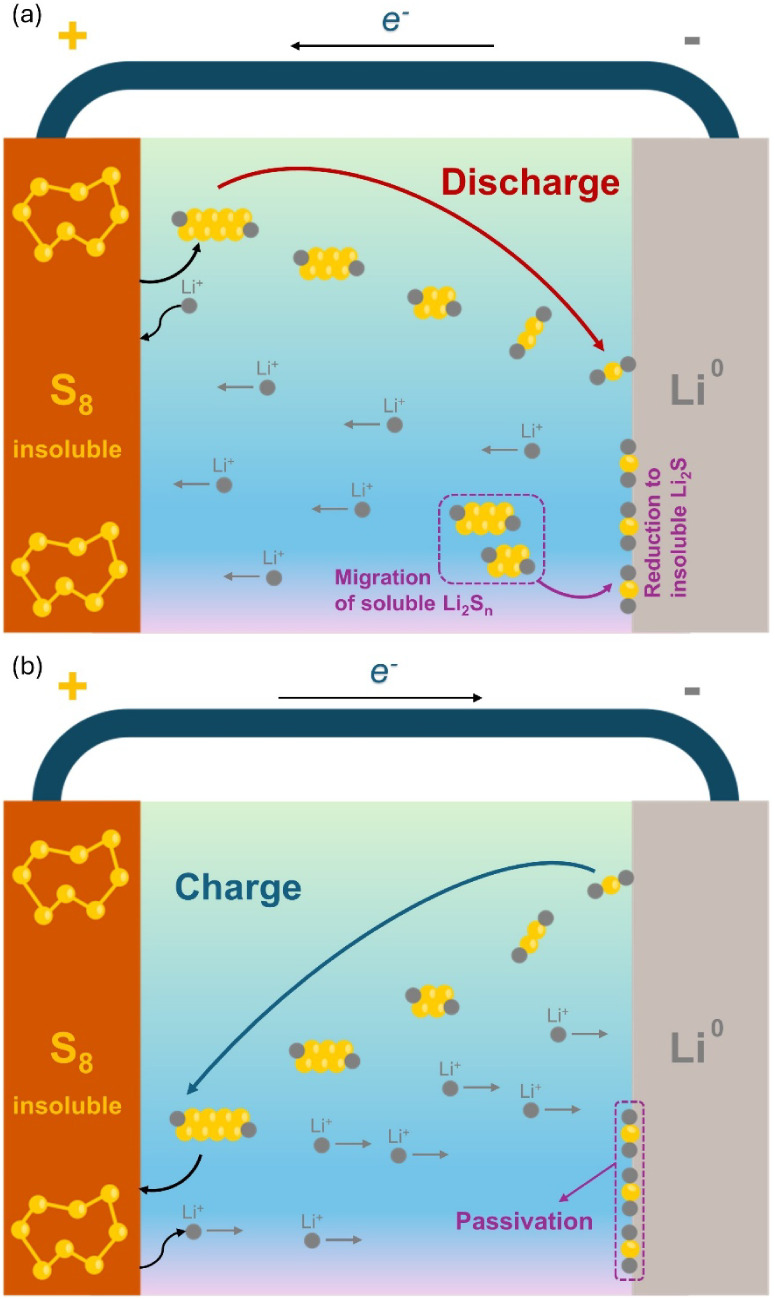
Representation of the shuttle effect and the deposition of insoluble LiPSs (Li_2_S and Li_2_S_2_) on the anode surface during the (a) discharge and (b) charge.

Hopefully, the drawbacks addressed above can be inhibited by encapsulating sulfur into conductive matrices (i.e., hosts), which can increase the cathode conductivity and suppress the volume expansion of sulfur. Furthermore, sulfur host materials can physically confine or chemically adsorb soluble LiPSs and inhibit their migration to the lithium anode. Then, several materials were investigated to increase the capacity retention of Li–S batteries.^34^ Indeed, numerous new materials and strategies have been developed aiming to improve the electrochemical performance of Li–S batteries (Figure 1), with the design of novel host materials emerging as one of the most promising trends.

Host materials that physically confine LiPSs include porous materials and materials with high specific surface area (SSA), which behave as physical barriers that encapsulate sulfur and inhibit the migration of LiPSs to the lithium anode, as the sulfur conversion reactions will happen within the host pores.^34^ It has been demonstrated that porous carbon-based materials with high specific surface area (SSA) can physically confine soluble LiPSs, such as activated carbon,^35^ carbon nanotubes (CNTs),^36,37^ graphene,^38,39^ and carbon fibers.^40,41^ On the other hand, some host materials can chemically adsorb LiPSs by bonding soluble LiPSs. In this context, polar hosts can catalyze the conversion of LiPSs to solid Li_2_S_2_ or Li_2_S, inhibiting their dissolution in the electrolyte. Despite the polysulfide shuttle suppression, polar hosts can contribute to increasing the slow redox kinetics of the insoluble Li_2_S_2_ and Li_2_S. Thus, the application of polar hosts can contribute to developing Li–S batteries with high values of reversible capacity and long lifespan, contributing to the design of commercial Li–S batteries.^4^

Among the several polar hosts, metal-free polar materials can contribute to the design of novel cathode materials for Li–S batteries, such as nitrogen­(N)-doped carbon hosts, which act as a conductive Lewis *base* catalyst to enhance the adsorption energy of long-chain LiPSs. Thus, composites comprising catalytic and porous carbon-based material can combine both physical confinement and chemical adsorption of soluble LiPSs and increase the conductivity of the electrode material.^42–44^ Besides, polymeric carbon nitrides can also contribute to the reversibility of Li–S batteries, as their surface presents electrostatic interaction with LiPSs.^4^

Catalytic metals used as sulfur hosts include noble metals, such as platinum (Pt),^45^ and gold (Au),^46^ nanoparticles. Transition metals provide a catalytic activity to suppress the shuttle effect, contributing to the adsorption of intermediate LiPSs (Li_2_S_*n*_, with *n* = 4–8) to the oxidation of Li_2_S_6_ to S_8_. However, some alternatives, such as noble metals, may present high costs, which inhibit their practical applications.^4^

In addition, metal oxides have been investigated as sulfur hosts, which have provided catalytic activity to chemically adsorb intermediate LiPSs and accelerate the slow oxidation redox kinetics of insoluble Li_2_S/Li_2_S_2_.^43,44^ Regarding the catalytic activity of metal oxides, some materials can convert soluble long-chain LiPSs (Li_2_S_*n*_, *n* > 4) into thiosulfate (S_2_O_3_^2–^), formed by the oxidation of these intermediate LiPSs. Then, LiPSs are chained into the S–S bond of thiosulfate, forming polythionate [O_3_S_2_-(S)_*n*−3_-S_2_O_3_]^2–^, and insoluble Li_2_S_2_ and Li_2_S.^42,47^ Furthermore, the chained S–S bonds present in the polythionate complex are electrochemically active during the Li–S battery’s conversion reactions, contributing to the adsorption of soluble LiPSs and accelerating the redox kinetics, which has been observed in a study conducted by Liang et al.^47^ The formation of thiosulfate and polythionate has been observed for several metal oxides, such as RuO_2_,^48^ V_2_O,^49^ V_2_O_3_,^50^ MnO_2_,^47^ and CeO_2_.^51^ Moreover, it has been reported that porous metal oxides with high SSA can physically confine soluble LiPSs, such as TiO_2._ ^52^

Furthermore, several metal sulfides have been investigated as sulfur hosts, which usually present a conductivity higher than metal oxides, and they can also accelerate the redox reactions and suppress the *shuttle effect*. Soluble LiPSs are usually electrostatically attracted by the polar surface of metal sulfides, as LiPSs are polar species, presenting positive (Li^+^) and negative (S_*n*_^2–^) charges.^53^ Among metal sulfides used as sulfur hosts, transition metal sulfides have been widely investigated, including Co_3_S_4_, TiS_2_, MoS_2_, VS_2_, and WS_2_. Similar to metal sulfides, metal nitrides can also be employed as sulfur hosts, as they present high conductivity and strong chemical interaction with LiPSs, acting as good catalysts, such as WN, VN, and TiN. Other metal-containing materials that provide the chemisorption of soluble LiPSs include materials such as metal selenides,^54^ metal phosphides,^55^ metal carbides,^56,57^ metal oxycarbides,^58^ metal oxycarbonitrides,^59^ composites,^60^ and many 2D materials.^61^ Thus, significant research has been performed on the design of cathode materials for Li–S batteries, but the search for materials that present low cost and can efficiently suppress the polysulfide shuttle is still in progress.^62,63^

Among other materials that have been investigated as sulfur hosts, coordination compounds such as metal–organic frameworks (MOFs) have been widely investigated, which can both chemically adsorb and physically confine LiPSs.^64,65^ Moreover, the recent use of PBAs as sulfur hosts has efficiently suppressed the polysulfide shuttle, providing high-capacity retention for Li–S batteries, which can contribute to the design of Li–S batteries with long cycling life.^8,17^ In fact, Prussian blue (PB) Fe_4_^3+^[Fe^2+^(CN)_6_]_3_·nH_2_O, a compound with a structure similar to MOFs, has demonstrated efficiency in adsorbing intermediate LiPSs due to its abundant Lewis active sites.^66^ Furthermore, PB analogues (PBAs) consist of several metal-containing compositions that can be fabricated by introducing other transition metal ions into the PB structure.^66,67^ In fact, PBAs have demonstrated that they can physically confine soluble LiPSs because of the adsorption sites of the metals that compose PBAs.^68^ The use of PBAs for this purpose has shown promising results, as well as their derivatives,^68^ which are further detailed in sections 4–5.

## 3 General Features and Synthesis Protocols of PBAs and Their Derivatives

Because of their straightforward production processes, cost-effectiveness, and environmentally friendly nature, associated with open porous and double transition metal connection structure, metal hexacyanoferrates exhibit a distinct advantage in battery application. These compounds can be represented by the Fe_4_^3+^[Fe^2+^(CN)_6_]_3_·nH_2_O minimal formula, called Prussian blue (PB), usually crystallizing in a face-centered cubic structure (space group Fm3m), where iron sites exhibit 2+ and 3+ oxidation states, regularly alternating and interconnected by cyanide ligands (−C≡N−). Additionally, Fe^2+^ ions are coordinated to carbon atoms, while Fe^3+^ ions are coordinated to nitrogen atoms, forming an octahedra. The compound’s electroneutrality is maintained by the Fe^2+^:Fe^3+^ ratio of approximately 3:4. Consequently, there are approximately 25% vacancies in the [Fe­(CN)_6_]^4–^ sites, which are filled by water molecules.^69^ There are compounds with structures similar to PB, known as PB analogues (PBAs), where other metals occupy the sites that belong to iron in the structure. Such compounds can be represented by the general formula A_*x*_M_a_[M_b_(CN)_6_]_1–*y*_ϒ_*y*_·nH_2_O, where M_a_ (N-coordinated site) and M_b_ (C-coordinated site) are transition metal ions, A is an alkali or alkaline earth metal ion, ϒ represents [M_b_(CN)_6_] vacancies, 0 ≤ *x* ≤ 2, and *y* < 1.

While these materials have certain advantages, they may also have limitations or challenges that need to be addressed, such as specific performance characteristics, compatibility with different types of metals, and scalability of production processes. Additionally, the field of battery technology is constantly evolving, and researchers are always exploring new materials and techniques to improve energy storage. In the case of Li–S batteries, the electrical isolation properties of sulfur, along with the expansion in volume during lithiation and the internal *shuttle effect* resulting from the dissolution of polysulfides, constitute the primary challenges associated with sulfur cathodes.^70^

The tuning of PBA properties is mostly achieved by the synthetic approach. For application in conventional metal-ion batteries, the principal objective of the synthesis is to obtain a material with high crystallinity and minimum water content, since defects in the structure can decrease the guest ion insertion capacity, slow ion kinetics, and attenuate the cycling performance.^67^ However, for Li–S devices, the presence of defects can originate in more active sites, increasing the interfacial adsorption of polysulfides.^71^ Moreover, due to the soft Lewis bases characteristic of polysulfide anions, a material containing Lewis acid metal sites (especially the soft ones) can strongly interact with S_*x*_^2^–, decreasing the diffusion of polysulfides out of the cathode and suppressing the *shuttling effect.* ^27^ The design of multimetal PBAs has recently emerged as a promising strategy for developing catalysts with synergistic effects for the conversion of LiPSs in Li–S batteries.^8^

The most used preparation approach for PBAs is the coprecipitation method, which is based on the reaction of [Fe­(CN)_6_]^4–^ or [Fe­(CN)_6_]^3–^ with a metal precursor salt. The variables influencing the synthesis encompass the concentration, duration, and temperature of the reaction solution, the method of mixing, and the incorporation of a chelating agent.^67^ Using this method, Chen et al.^72^ synthesized S–Na_2_Co­[Fe­(CN)_6_]/reduced graphene oxide (rGO) composites and applied as a cathode for Li–S, combining the larger transmission channel of the hexacyanoferrate for the insertion/extraction of Li^+^ and the absorption/confinement of polysulfide proportioned by the carbon nanostructure.^72^ Since Prussian blue and its analogues have sulfur/polysulfide adsorption properties compared to carbon structures, and these properties are strongly related to the presence of different metals in the structure, the synthesis of PBAs with more metallic species in the structure is desired. Feng et al.^73^ prepared a PBA material, K_1.37_(Ni_0.28_Mn_0.72_)­[Fe­(CN)_6_]_0.83_·2.38H_2_O (M_a_ site containing NiMn), coated with polypyrrole as a cathode for Li–S batteries.^73^ The addition of Ni^2+^ in the structure proportionates a smaller distortion of the MnN_6_ unit, decreasing the Jahn–Teller effect, reducing the structural stress during cycles, and consequently, a higher stability of the material is obtained. In conjunction, the coating of polypyrrole improved the electron conductivity of the material surface and acted as a physical barrier to reduce the shuttle effect of the electrode. Du et al.^68^ showed that a polypyrrole-enveloped PBA containing FeCoNi promotes a multimetal synergistic effect for the increasing of Li_2_S_4_ absorption and cycling performance of the electrode.^68^

Although the vast majority of published studies report the preparation of PBAs containing two or three metal ions, including Fe (referred to here as binary and ternary PBAs, respectively), there is a growing trend toward the design of more complex PBAs, particularly by increasing the entropy of the M_a_ site. For instance, incorporating three or four different metal ions in equimolar proportions at the M_a_ site of PBAs enables the design of promising ME-PBAs. In a related study, Liu et al.^74^ reported the coexistence of entropy-regulation transition metal elements, such as Mn, Co, Ni, and Cu ions, positioned at the N-coordinated M_a_ sites within the ME-PBA framework, while Fe remains at the C-coordinated M_b_ sites. Analogously, the HE-PBAs characterized by the inclusion of five or more metal elements (within the same metal site) forming a unified lattice with random occupancy hold the potential to yield exceptional structural materials. So, it is plausible to posit that integrating the high-entropy concept into PBAs offers significant prospects for enhancing their conductivity and catalytic efficiency.^75^ Presently, only a restricted set of HE-PBAs has been successfully synthesized, primarily due to the challenges associated with blending elements possessing markedly distinct chemical and physical characteristics, coupled with limitations in cooling rates. Furthermore, reducing HE-PBAs to the nanoscale poses a great challenge, particularly when employing conventional techniques. In the context of PBAs, the selection of metal cations with similar hexacoordinated ion radii is crucial to diminish lattice barriers. Therefore, the advancement of synthesis methodologies that enable precise control over elemental composition, particle size, and phase holds the potential to introduce a novel array of nanostructures with unparalleled functionalities. Jiang et al.^76^ prepared, at room temperature, using a combination of mechanochemistry and wet-chemistry methods, five different HE-PBAs with a chemical formula of KMFe­(CN)_6_, where M (N-coordinated site, M_a_) represents five different metal cations (Mg^2+^, Mn^2+^, Fe^2+^, Co^2+^, Ni^2+^, and Cu^2+^).^76^ In the first step, the authors used ball milling to prepare a fine mixture with low crystallinity, with the consequent removal of K^+^ and Cl^–^ ions through water rinsing to obtain crystalline HE-PBAs (Figure 3). Some studies conducted by Ma et al.^77,78^ applied the high-entropy concept to prepare manganese hexacyanoferrates.^77,78^ In a recent work conducted by Ma et al.^77^, the authors obtained a material containing four metal ions with a chemical formula Na_*x*_Mn_0.4_Fe_0.15_Ni_0.15_Cu_0.15_Co_0.15_[Fe­(CN)_6_]. The work used the coprecipitation method to obtain cubic particles with an average size of 1 μm.^77^ Moreover, Huang et al.^79^ used a coprecipitation method to prepare a polycrystalline HE-PBA on a kilogram scale. Sodium citrate was used as a chelating and sodium-rich agent, slowing down the release of transition metal ions, increasing the formation of a low-vacancy structure with the chemical formula of Na_1.70_Fe_0.2_Mn_0.2_Co_0.2_Ni_0.2_Cu_0.2_[Fe­(CN)_6_]_0.98_□_0.02_·2.35H_2_O.^79^

**Figure 3:**
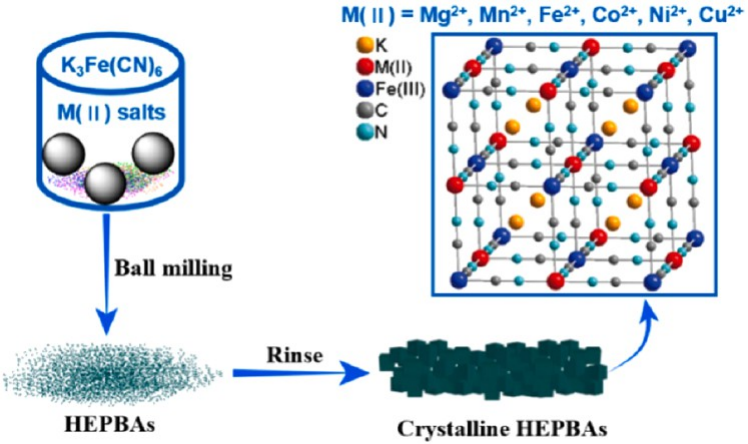
Diagrammatic depiction of the formation of HE-PBAs through ball milling and washing at ambient temperature. Reproduced with permission from ref.^76^ Copyright 2020, Elsevier Ltd. All rights reserved.

While HE-PBAs hold promise for developing superior structural materials with enhanced performance in storage applications, there is currently a lack of research papers investigating their potential use in Li–S batteries. In addition, although PBAs present the relevant properties mentioned above for the application as cathodes in Li–S batteries, there is the possibility to increase the availability of metals in the material surface and the electroconductivity through the design of PBA-derived materials.^80,81^ In fact, the production of PBA derivatives has resulted in promising advances in catalysts for several emerging energy applications, including Li–S batteries.

The synthesis of PBA-derived materials can generally be divided into three main stages: (i) pretreatment, (ii) derivatization, and (iii) post-treatment, as summarized in Figure 4 (more details can be found in the review by Bornamehr et al.^82^ The derivatization is the crucial step, defined here as the process that transforms a PBA into other materials. Pretreatment is often conducted before derivatization, and in some cases, a post-treatment follows to finalize the overall procedure. Each of these stages is strategically selected to impart specific characteristics to the material, such as its morphological, physical, or chemical properties, which enhance its performance for the intended application. These steps can be executed through either a single process or a combination of processes, with thermal treatment being the most used due to its versatility. For instance, by precisely controlling factors such as temperature and atmosphere (often involving a reactive carrier gas), various pathways can be tailored to achieve the desired transformations.^82^

**Figure 4:**
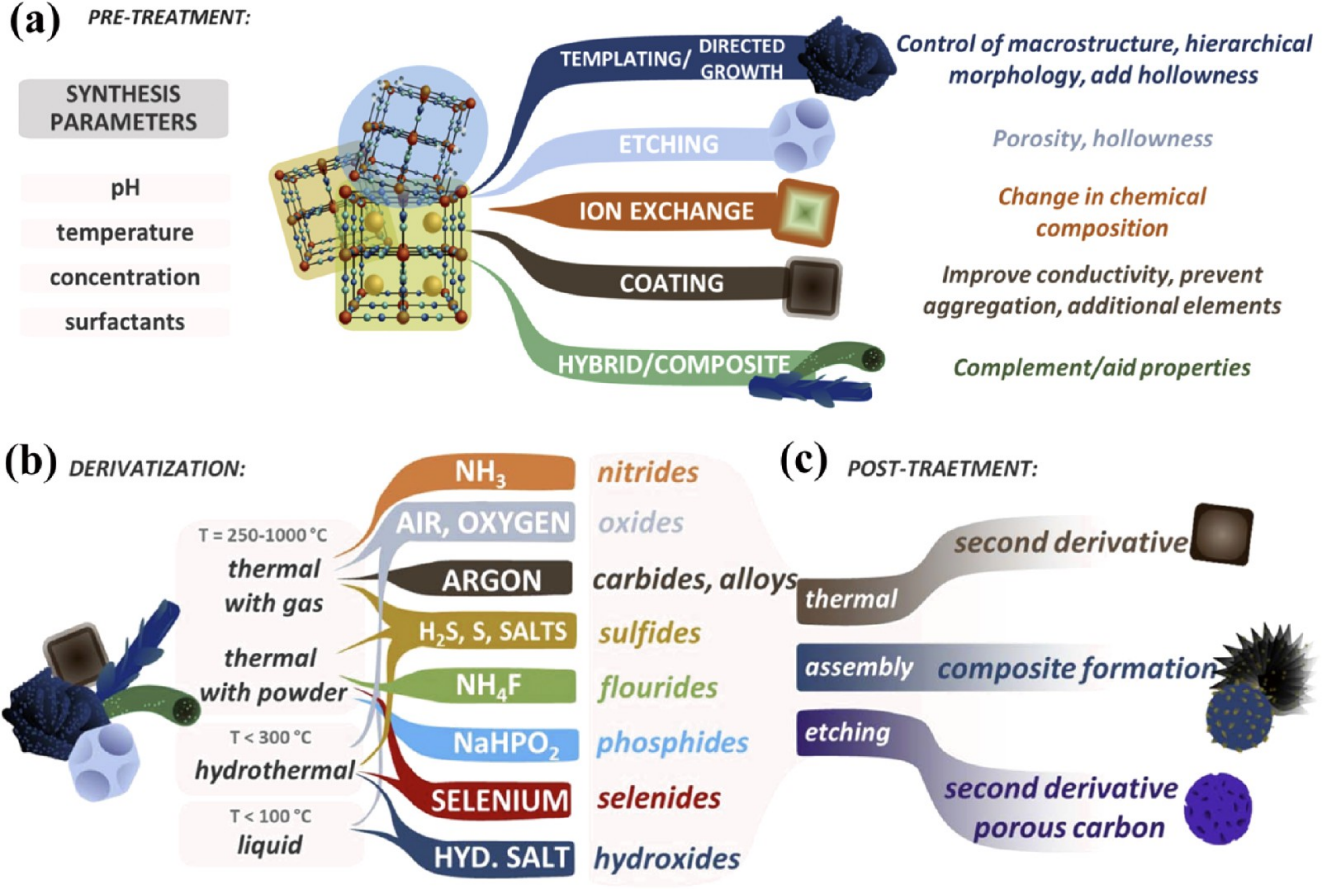
(a) Approaches to modify PBA particles before derivatization, aiming for similar target outcomes. These modifications are conducted alongside synthesis parameter adjustments, including pH, temperature, salt concentration, and surfactant presence. Summary of common compounds derived from PBAs and the typical derivatization pathway (b) and post-treatments (c). Reproduced with permission from ref. 82. Copyright 2023, The Royal Society of Chemistry.

The most prepared PBA-derived materials for Li–S batteries are CoFe-containing structures. The literature explores the synthesis of materials in different conditions of atmosphere, temperature, and time of pyrolysis, such as carbon/CoFe nanocubes (N_2_, 500 °C, 3 h),^83^ C/CoFe_2_O_4_ nanocages (air, 400 °C, 2 h) with subsequent annealing under argon at 600 °C for 2 h,^70^ hollow Co_*x*_Fe_3–x_O_4_ nanocages (O_2_, 350 °C, 6 h),^84^ N-doped porous carbon/CoFe (air, 300 °C, 2 h, and post, argon, 1000 °C, 2 h),^84^ flower-like Co/phosphosulfide/graphene (Ar, 500 °C, 1 h),^85^ and a mixture of CoS_2_ nanoparticle-embedded N-doped carbon nanoboxes (Ar, 350 °C, 2 h) and Fe/carbon nanotubes.^86^ After the pyrolysis step, generally, the metallic composites are submitted to sulfur incorporation, usually using a melt-diffusion method in an argon atmosphere at 155 °C for 10–12 h.

As described before, the temperature is an important experimental parameter for the preparation of PBA-derived metal-based materials. Studies conducted by Qi et al.^87^ and Zhu et al.^88^ have reported synthesized CoFe and NiCo nanoparticles on an N-doped carbon matrix and studied different annealing temperatures (400–900 °C). The distribution of metal nanoparticles and N heteroatoms improved the sulfur electrochemistry and led to superior conductivity. Li et al.^89^ prepared a hollow porous spinel NiCo_2_S_4_–S composite by using Ni_5/3_Co_4/3_[Co­(CN)_6_]_2_ PBA as the precursor, which was carbonized in H_2_S flow at 350 °C.^89^ This material proportioned effective confinement and labile kinetics of polysulfides. The approach used by Wang et al.^90^ generated carbon nanotubes filled with Fe/CeO_2_.^90^ In this paper, the authors utilized Ce­[Fe­(CN)_6_] and melamine as the precursors, obtaining different series of marine organism-like architectures according to the ratio of the precursors, which were annealed at 900 °C in an N_2_ atmosphere.

In the work of Song et al.^91^ a Fe–Ni–P@nitrogen-doped carbon nanocomposite was prepared by heating the respective PBA to 300 °C for 2 h under an Ar atmosphere (Figure 5). Besides the increase in the adsorption of LiPSs led by the metal-containing particles, the N-doped carbon derived from the CN-ligand of the PBA structure increased the conductivity of the electrode.

**Figure 5:**
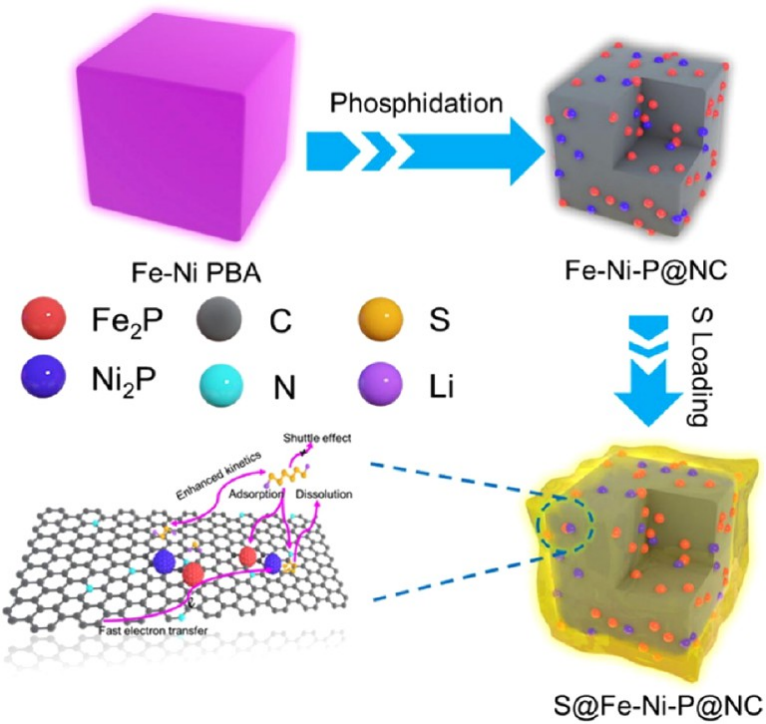
Schematic representation of the preparation of S@Fe–Ni–P@NC composites. Reproduced with permission from ref. 91. Copyright 2021, Elsevier Inc. All rights reserved.

The use of PBAs as precursors for the preparation of ternary compounds has emerged as an effective strategy to combine multiple conversion sites and thus increase both surface adsorption and charge transfer in the electrochemical conversion of Li–S species. Yang et al.^92^ prepared a FeCoNi phosphide compound into carbon architectures derived from the respective PBA, which was annealed at 350 °C for 3 h under an air atmosphere.^92^

While enhancing the conductivity of PBAs by transforming them into metal oxides or other nanoparticles can yield superior results compared to PBAs in their original state, this approach is suboptimal due to the possibility of release of highly toxic cyanide during the pyrolysis of PBAs. PBAs possess ample active sites inherently, and enhancing their conductivity can enable them to function directly as electrocatalysts.^79^

## 4 Low-, Medium-, and High-Entropy PBAs for Li–S Batteries

In order to efficiently suppress the polysulfide shuttle, PBAs have been investigated as sulfur host materials, as they present a plethora of adsorption sites to chemically adsorb LiPSs and suppress the *shuttle effect*, especially those based on multimetal-containing compounds. Furthermore, PBAs are porous coordination polymer materials that can physically confine LiPSs, presenting an architecture similar to MOFs.^68,93^ Moreover, PBAs can be classified as low-entropy (LE-PBAs), medium-entropy (ME-PBAs), and high-entropy (HE-PBAs) according to the number of elements comprising their structure. In this context, the configurational entropy (*ΔS*_*config*_) per mole of PBAs can be expressed by eq 1, where R consists of the gas constant (8.314 J mol^–1^ K^–1^), and *x*_*i*_ and *x*_*j*_ consist of the mole fractions for the cation and anion sites, respectively. *N* is assigned to the number of elements present in the material. In this context, HE-PBAs (i.e., comprising five or more elements) should present a Δ*S*_config_ higher than 1.5 R. In contrast, ME-PBAs (i.e., comprising typically three or four elements) should present a Δ*S*_config_ from 1.0 to 1.5 R, and LE-PBAs (i.e., comprising typically two or fewer elements) should present a Δ*S*_config_ lower than 1.0 R (Figure 6).^8,94^

1 $$\Delta S_{\mathrm{config}}=-R\left[{\left(\sum_{i=1}^{N}x_{i}\;\ln x_{i}\right)}_{\mathrm{cation‐site}}+{\left(\sum_{j=1}^{M}x_{j}\;\ln x_{j}\right)}_{\mathrm{anion‐site}}\right]$$

**Figure 6:**
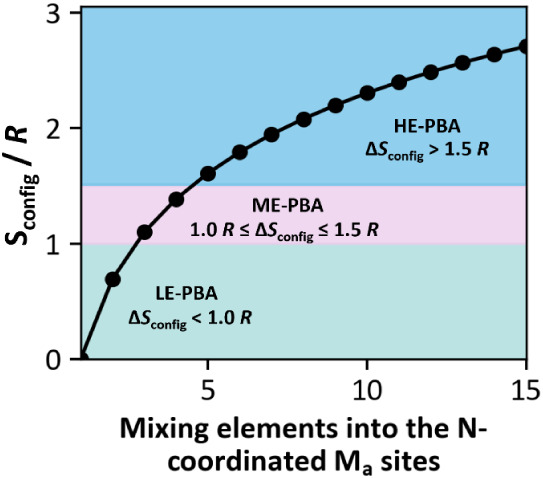
Configurational entropy values as a function of the number of metal ions incorporated at the N-coordinated M_a_ site, along with the visual separation between LE-, ME-, and HE-PBAs based on the entropy values calculated using eq[Disp-formula eq1] ref 95. Copyright 2022, The American Association for the Advancement of Science.

Despite the straightforward definitions presented above, misunderstandings are frequently found and propagated in the literature, particularly when the concept of high entropy is applied to materials featuring more than one metal site, such as PBAs, spinel oxides, perovskites, etc. In the case of PBAs, typically only the M_a_ site (N-coordinated) is substituted, while Fe occupies the C-coordinated M_b_ sites. In this context, to be considered a HE-PBA, the N-coordinated site must be bonded to at least five metal ions in equimolar or near-equimolar proportions. It is essential to consider the calculation of configurational entropy to avoid errors, as not all multimetal PBAs with a total of five metal ions or with five metal ions at the N-coordinated site are necessarily HE-PBAs. For instance, the maximum entropy of a multimetal PBA with four metal ions at the N-coordinated site is approximately 1.386*R* (<1.5*R* for an HE-PBA), meaning it cannot be classified as an HE-PBA. Additionally, many compounds, rich in a specific metal ion, should also generally not be considered HEMs, even if they contain five metal ions. In such cases, the calculation of configurational entropy is essential to avoid errors and the propagation of misunderstandings. Moreover, these considerations should also be applied to LE-PBAs and ME-PBAs, where the calculation of configurational entropy should serve as a guide to prevent errors.

Considering the above-mentioned definitions, several PBAs have been investigated as sulfur hosts for Li–S batteries. For instance, in a study conducted by Du et al.^68^ the authors investigated the performance of a ternary PBA-based sulfur host material (i.e., LE-PBA) containing C-coordinated Fe site, and N-coordinated Co and Ni sites, named FeCoNi-PBA. The performance of the ternary FeCoNi-PBA was compared with binary FeCo- and FeNi-PBAs, which have shown inferior results (Table 1). Thus, the ternary host (FeCoNi-PBA) presented more active sites to chemically adsorb intermediate LiPSs than binary PBAs counterparts (FeCo- and FeNi-PBA), resulting from its synergistic effect, and it was observed in the LiPSs adsorption test, UV–vis spectroscopy of the adsorbed solution, and XPS.^68^ Furthermore, density functional theory (DFT) was conducted to unveil the interactions between Li_2_S_4_ with FeNi-, FeCo-, and FeCoNi-PBAs, which presented adsorption energy values of 1.043, 0.893, and 2.636 eV, respectively. Thus, DFT confirmed that ternary FeCoNi-PBA presented more adsorption sites than the binary PBAs, which favored its performance as a host material in Li–S batteries.^68^ The better performance of ternary compounds when compared to analogues with lower configurational entropy was also demonstrated in other works.^68,73^

**Table 1:** Performance of PBA Materials Applied as Cathodes for Li–S Batteries

**Material**	**Metals**	**Sulfur content (wt %)**	**Sulfur loading (mg cm** ^**–2**^)	**Reversible Capacity (mAh g** ^**–1**^ **)/Capacity retention (%)**	**Initial Capacity (mAh g** ^**–1**^)	**WVW (V vs Li/Li** ^**+**^)	**ref.**
Mn-PBA@S	MnFe	-	-	577/∼64.9% after 100 cycles @ 0.2C	889 @ 0.2C	1.5 – 2.8	^73^
3-MnNi-PBA@S (Ni:Mn ratio of 3:7)	MnNiFe	49	-	798/∼92.3% after 100 cycles @ 0.2C	865 @ 0.2C		
3-MnNi-PBA@S@PPy (Ni:Mn ratio of 3:7)	MnNiFe	41	2.5–4.0	908/∼70.7% after 100 cycles @ 0.1C	1285 @ 0.1C		
				518/∼60% after 500 cycles @ 0.5C	842 @ 0.5C		
5-MnNi-PBA@S (Ni:Mn ratio of 5:5)	MnNiFe	49	-	619.3/∼86.2% after 100 cycles @ 0.2C	718.1 @ 0.2C		
FeCoNi-PBA-S-PPy	CoNiFe	41	∼ 1.2–1.4	703.6/∼51.4% after 100 cycles @ 0.1C	1369.3 @ 0.1C	1.7 – 2.7	^68^
				490/- after 200 cycles @ 0.5C	-		
FeCoNi-PBA-S	CoNiFe	48		451.2/∼36.5% after 100 cycles @ 0.1C	1234.7 @ 0.1C		
				360.1/- after 200 cycles @ 0.5C	-		
FeNi-PBA-S	FeNi	50		206.9/∼15.9% after 100 cycles @ 0.1C	1303.2 @ 0.1C		
				185.8/- after 200 cycles @ 0.5C	-		
FeCo PBA-S	FeCo	49		370.1/∼36.0% after 100 cycles @ 0.1C	1029.4 @ 0.1C		
				250.4/- after 200 cycles @ 0.5C	-		
PB-S	Fe	-		231.3/- after 100 cycles @ 0.1C	-		
				171.2/- after 200 cycles @ 0.5C	-		
S@C–Co–PBA@CCNB-SAFe-MWCNT	CoFe	38.6	1	550/∼78.6% after 500 cycles @ 1C	700 @ 1C	1.7 – 2.8	^86^
				-	538 @ 2C		
S@Na_2_Fe[Fe(CN)_6_]	Fe	∼55.4	-	1006/∼90.9% after 19 cycles @ 0.2C	1107 @ 0.2C	1.7 – 2.7	^27^
			-	891/∼87.5% after 42 cycles @ 0.5C	1018 @ 0.5C		
			-	782/∼88.7% after 86 cycles @ 1C	882 @ 1C		
			-	690/∼88.5% after 100 cycles @ 2C	780 @ 2C		
			-	596/∼83.1% after 103 cycles @ 5C	717 @ 5C		
S@Na_2_Fe[Fe(CN)_6_]@poly(3,4-ethylenedioxythiophene)	Fe	∼80	-	1101/∼85.3% after 100 cycles @ 0.1C	1291 @ 0.1C	1.7 – 2.7	
			-	770/∼76.9% after 100 cycles @ 1C	1001 @ 1C		
			-	697/∼85.1% after 100 cycles @ 2C	819 @ 2C		
			-	544/∼79.8% after 200 cycles @ 5C	683 @ 5C		
S@Na_2_Co[Fe(CN)_6_]@rGO	FeCo	74	0.8	-	1163 @ 0.1C	1.8 – 2.8	^72^
				858/∼93.5% after 100 cycles @ 0.5C	918 @ 0.5C		
				520/∼83.9% after 100 cycles @ 0.5C	620 @0.5 C		
S@K_3_[NiFe(CN)_6_]	NiFe	70	-	339.5/∼30.1% after 200 cycles @ 0.1C	1129 @ 0.1C	1.7 – 2.7	^17^
S@ K_3_[NiCuFe(CN)_6_]	NiCuFe			389.8/∼33.3% after 200 cycles @ 0.1C	1169.1 @ 0.1C		
S@ K_3_ [CoNiCuFe(CN)_6_]	CoNiCuFe			467/∼38.3% after 200 cycles @ 0.1C	1218.3 @ 0.1C		
S@K_3_[CoNiCuMnZnFe(CN)_6_]	CoNiCuMnZnFe			570.9/∼42.7% after 200 cycles @ 0.1C	1335.6 @ 0.1C		
Co_0.4_Ni_0.4_[Fe(CN)_6_]_2_	CoNiFe	-	1.0	386.4/- after 200 cycles @ 0.1C	-	1.7 – 2.7	^98^
Mn_0.4_Co_0.4_Ni_0.4_Cu_0.4_Zn_0.4_[Fe(CN)_6_]_2_	MnCoNiCuZnFe			705.4/∼65.5% after 200 cycles @ 0.1C	1076.4 @ 0.1C		

With the advancement of ternary PBA development, emerging trends have been reported to further enhance the performance of these multimetal catalysts. For instance, the approach of coating the surface of PBAs with conductive polymers is an interesting alternative to increase the physical confinement of LiPSs (forming a physical barrier), and consequently extend their cyclability, as it has been widely investigated in other materials.^96^ In this context, Du et al.^68^ reported that the encapsulation of ternary PBAs within the polypyrrole (PPy) layer can be achieved by water-phase chemical oxidative polymerization, which can be conducted by immersing the sulfurized PBA in an aqueous solution, resulting in the *in situ* coating of the polymer.^68^ This process is detailed in Figure 7a, which evidence the polymer coating procedure of a sulfurized ternary PBA. The morphology of the PBAs has been investigated by SEM and TEM, and it showed that the FeCoNi-PBA nanocubes presented dimensions of ≈ 200 nm (Figure 7b and e). Furthermore, the TEM image of the sulfurized FeCoNi-PBA nanocubes (FeCoNi-PBA-S) indicates that sulfur has been diffused into the structure of the FeCoNi-PBA nanocubes, and their volume was not significantly altered (Figure 7c). Moreover, the TEM results of the FeCoNi-PBA-S with PPy (FeCoNi PBA-S-PPy) evidenced that the FeCoNi-PBA-S nanocubes were coated by a PPy layer, which presented a thickness of ≈ 40 nm (Figure 7d). Finally, the elemental mapping obtained from STEM-EDS confirmed the homogeneous distribution of Fe, Co, and Ni in the ternary PBA nanocubes, as well as the distribution of PPy and sulfur (Figure 7h). Furthermore, the Li–S battery comprising the FeCoNi-PBA-S-PPy cathode showed capacity retention of ∼ 51.4% after 100 cycles at 0.1C (reversible capacity of 703.6 mAh g^–1^), which has been significantly superior to the values provided by the system comprising the pristine ternary FeCoNi-PBA-S and binary-based cathodes (i.e., FeNi- and FeCo-PBAs), as evidenced in Table 1, confirming that the PPy coating procedure should be considered while designing novel sulfur hosts (Figure 7i–j). Furthermore, EIS results have evidenced a minimized resistance for the cell comprising the FeCoNi-PBA-S-PPy cathode, favoring ion transportation and consequently enabling higher capacity values.^68^ Moreover, DFT calculations were used to investigate the interactions between Li_2_S_4_ and PBAs. The ternary FeCoNi-PBA provides multiple adsorption sites, forming bonds with Li_2_S_4_. The adsorption energies for binary FeCo-PBA, FeNi-PBA, and ternary FeCoNi-PBA are 0.893, 1.043, and 2.636 eV, respectively, with FeCoNi-PBA showing the strongest interaction (Figure 7k–m). This enhanced adsorption, due to the synergistic effect of multiple metals, improves Li_2_S_4_ retention and cycling performance. The ability of PPy to adsorb soluble LiPSs has been previously evidenced, particularly by *operando* Raman spectroscopy and time-dependent UV–vis spectroscopy,^97^ which confirms that PPy can be used to coat PBAs, contributing to the design of potential sulfur hosts.

**Figure 7:**
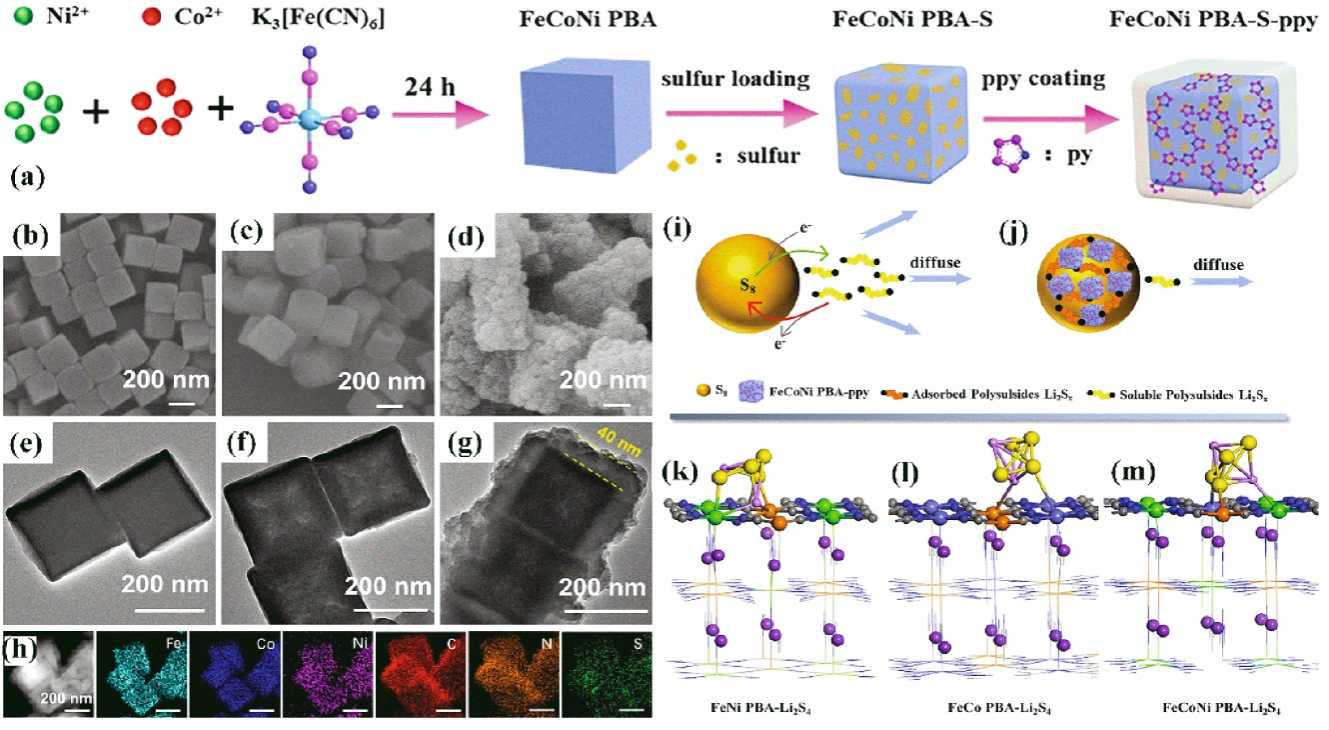
(a) Representation of the polymer coating procedure in a sulfurized PBA. (b) SEM and TEM images of the (b,e) pristine FeCoNi-PBA, (c,f) FeCoNi-PBA-S, and (d,g) FeCoNi-PBA-S-PPy. (h) STEM and elemental mapping of the FeCoNi-PBA-S-PPy. Diagram illustrating the adsorption process of polysulfides on FeCoNi–PBA–PPy: (i) cathode with pure sulfur, (j) FeCoNi-PBA-S-PPy cathode. Adsorption configurations of the Li_2_S_4_ molecule on the (100) surfaces of (k) FeNi-PBA, (l) FeCo-PBA, and (m) FeCoNi-PBA. Color legend: orange – Fe; green – Ni; light blue – Co; yellow – S; magenta – Li; purple – K; gray – C; blue – N. Reproduced with permission from ref. 68. Copyright 2021, Elsevier.

More significant effects of conductive polymers in ternary PBA were observed in the work of Feng and colleagues.^73^ In more detail, a ternary MnNi-PBA precursor was prepared through a one-step self-assembly precipitation process (although the authors do not explicitly mention the Fe ions in the compound name, such ions are part of the PBA structure). Sulfur was introduced into the porous structure and surface of the ternary MnNi-PBA via a melting diffusion technique. Then, the conductive polymer PPy was coated *in situ* in an aqueous solution using Fe^3+^ as an oxidizing agent (Figure 8a–c), which resulted in the electrode material called 3-MnNi-PBA@S@PPy, where 3 is related to the dosage of Ni and Mn sulfate. The 3-MnNi-PBA@S@PPy composite demonstrates strong chemisorption of LiPSs and outstanding catalytic activity, effectively anchoring LiPSs and accelerating their conversion. The ternary 3-MnNi-PBA surface reacts spontaneously with LiPSs, forming intermediate thiosulfate salts that enhance conversion kinetics. As a cathode material for lithium–sulfur batteries (LSBs), this composite delivers a high initial capacity of 1285 mAh g^–1^ at 0.1C, with remarkable cycling stability, maintains 518 mA h^–1^ g over 500 cycles at 0.5C, with a great capacity retention of ∼ 92.3% which is superior the ternary 1-MnNi-PBA@S (∼ 91.6%) and binary Mn-PBA@S (∼ 64.9%) without PPy. In general, the ternary MnNi-PBA framework enhances structural stability by mitigating internal stress and increasing the availability of active sites. Unlike conventional PBA materials, 3-MnNi-PBA and Mn-PBA react *in situ* with polysulfides, forming insoluble thiosulfates that link long-chain LiPSs and convert them into shorter-chain species. This transformation reduces the LiPS concentration in the liquid phase and significantly suppresses the shuttle effect. Additionally, the PPy coating improves electrical conductivity and acts as a barrier, further restricting LiPS diffusion. Experimental data and theoretical calculations confirm that the composite enhances LiPS conversion kinetics and reduces polarization during sulfur redox reactions. First-principles calculations indicate that 3-MnNi-PBA has high adsorption energies for LiPSs (2.31, 1.15, 1.35, 2.07, and 2.64 eV for Li_2_S_8_, Li_2_S_6_, Li_2_S_4_, Li_2_S_2_, and Li_2_S, respectively), aligning well with static adsorption experiments.

**Figure 8:**
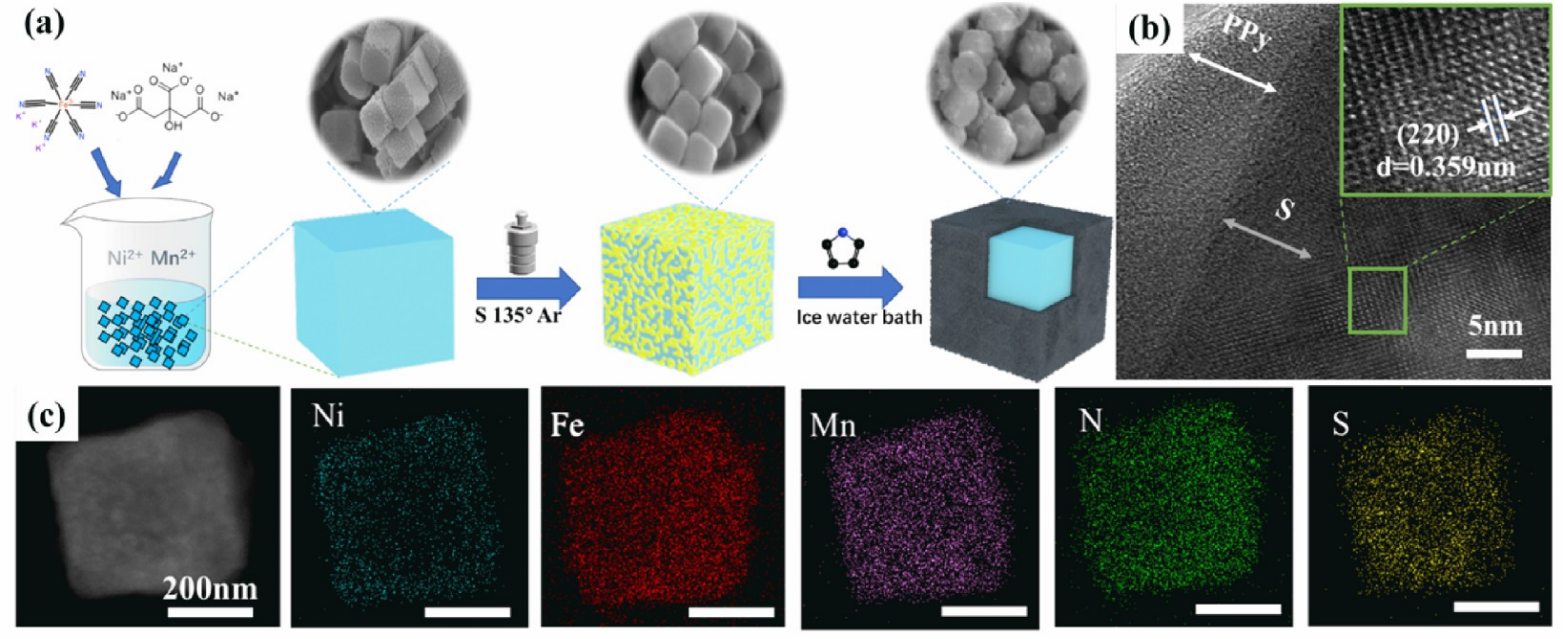
(a) Schematic representation of the MnNi-PBA@S@PPy composite synthesis process. (b) TEM images of ternary MnNi-PBA@S@PPy highlighting the PPy coating. The inset in (b) shows an HRTEM image of the highlighted green area. (c) EDS elemental mapping of MnNi-PBA@S@PPy. Reproduced with permission from ref. 73. Copyright 2022, Elsevier Ltd. All rights reserved.

Although some studies have used PPy to coat low-entropy PBAs and increase their conductivity^68,73^ other conductive polymers have been investigated to increase the conductivity of PBAs, such as poly­(3,4-ethylenedioxythiophene) (PEDOT), which was investigated in a study conducted by Su et al.,^27^ where they reported a Na_2_Fe­[Fe­(CN)_6_] coated with PEDOT. This approach increased the system’s reversible capacity from 763 to 1101 mAh g^–1^ at 0.1C after 100 cycles (Table 1). Besides, the S@Na_2_Fe­[Fe­(CN)_6_]@PEDOT composite electrode exhibited an outstanding reversible capacity of 544 mA h g^–1^ after 200 cycles at a high current rate of 5C, demonstrating its potential as a high-power electrode with remarkable capacity retention. In fact, the PEDOT-coating approach not only resulted in higher capacity but also enabled the design of electrodes with high power density (considering similar sulfur content). Therefore, other polymers can be investigated to contribute to the conductivity of PBAs by polymer-coating procedures, which can increase the chemical adsorption of LiPSs and provide improved electrochemical properties for Li–S batteries.

Although the studies mentioned above primarily focus on the application of LE-PBAs containing only two metal ions at the N-coordinated site, HE-PBAs are also an interesting alternative for sulfur hosts in Li–S batteries. One study conducted by Shen et al.^98^ used a HE-PBA, which consisted of five metals in its structure, particularly Mn^2+^, Co^2+^, Ni^2+^, Cu^2+^, and Zn^2+^, forming (Mn_0.4_Co_0.4_Ni_0.4_Cu_0.4_Zn_0.4_[Fe­(CN)_6_]_2_) cubic structures, which have been prepared by the coprecipitation in aqueous solution (Figure 9a), and the further sulfurization of the HE-PBA has been conducted by melt-diffusion procedure. The presence of multiple metals and their uniform distribution in the HE-PBA nanocubes was confirmed by EDS-STEM elemental mapping, as displayed in Figure 9b. The sulfurized HE-PBA has been used as a cathode for Li–S batteries, and its performance has been compared with the results of a sulfurized LE-PBA, which consisted of CoFe-PBA. The HE-PBA provided superior capacity values than the LE-PBA at different current densities (Figure 9c). Furthermore, the performance of the HE-PBA and LE-PBA cathodes was investigated by cycling the cells at 0.1C for 200 cycles (Figure 9d), which resulted in the reversible capacities of 705.4 mAh g^–1^ and 386.4 mAh g^–1^. Furthermore, a longer cyclability test was performed by cycling the cell at 1C for 850 cycles, which resulted in a reversible capacity of 417.6 mAh g^–1^ for the HE-PBA. In contrast, the LE-PBA resulted in a capacity of 205.6 mAh g^–1^ after only 200 cycles (Figure 9e).

**Figure 9:**
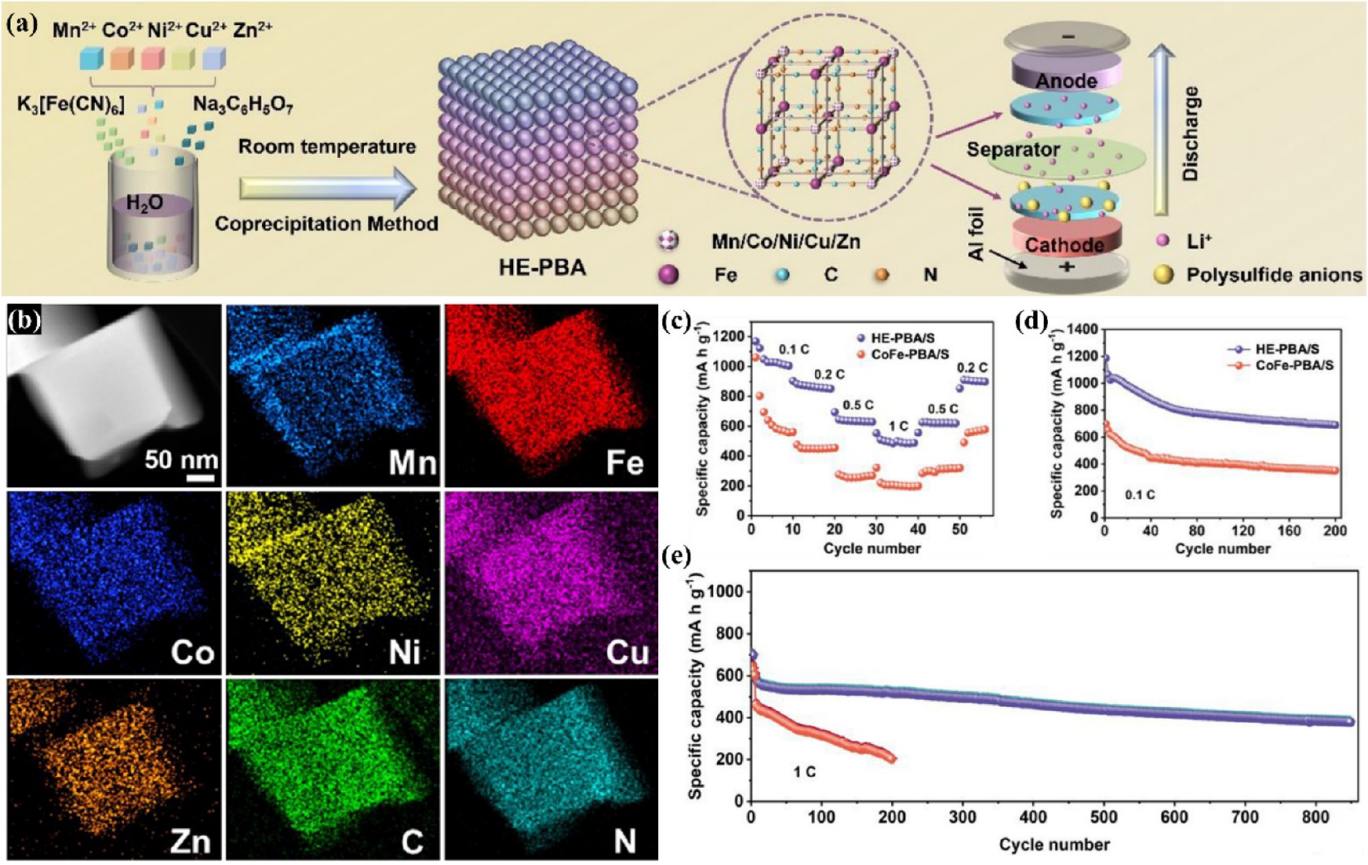
(a) Representation of the preparation of the HE-PBA Mn_0.4_Co_0.4_Ni_0.4_Cu_0.4_Zn_0.4_[Fe­(CN)_6_]_2_ electrode; (b) STEM and STEM-EDS elemental mapping of Mn, Fe, Co, Ni, Cu, Zn, C, and N for the HE-PBA; (c) rate capability; (d) cycling performance at 0.1C; and (e) cycling performance at 1C. Reproduced with permission from ref 98. Copyright 2024, Royal Society of Chemistry.

Furthermore, the reversible capacity provided by the study conducted by Shen et al.^98^ has shown a superior reversible capacity than previous HE-PBA hosts, as reported by Du et al.^17^ which reported the capacity provided by the CoNiCuMnZnFe- and CoNiCuFe-containing PBA hosts, and provided the reversible capacities of 570.9 mAh g^–1^ and 467 mAh g^–1^, respectively, after 200 cycles at 0.1C (Table 1). The Mn_0.4_Co_0.4_Ni_0.4_Cu_0.4_Zn_0.4_[Fe­(CN)_6_]_2_-based cathode designed by Shen et al.^98^ delivered capacity retention of ∼ 65.5%, which is higher than the CoNiCuMnZnFe-PBA-containing cathode (∼42.7%) after 200 cycles at 0.1C. Otherwise, the study conducted by Du et al.^17^ evidenced that HE-PBAs sulfur hosts can provide superior performance than ME- and LE-PBA-based cathodes. One plausible reason for HEMs and MEMs presenting superior adsorption of LiPSs is because of their multimetal synergistic effect, which can increase the adsorption of LiPSs.^99^ Moreover, HEMs present more metals dispersed, compared to LEMs and MEMs, which can indicate more active sites to anchor LiPSs, facilitate the oxidation of LiPSs, and increase the slow redox kinetics of Li_2_S.^100^

The application of PBAs with carbon-based materials has also been observed for sulfur hosts, which can be referred to as carbon-composite materials. Carbon-containing composite materials provide high adsorption of LiPSs, as they can combine the physical confinement of LiPSs through the pores of the free carbon framework, with the chemical adsorption provided by the polar bonds of the metals present in the host material.^101–103^ This strategy has been observed for PBA-based cathodes, including different carbon materials, such as reduced graphene oxide (rGO)^72^ and other carbon nanomaterials.^86^ One study conducted by Chen et al.^72^ evidenced that the procedure of coating a binary Na_2_CoFe­(CN)_6_ (named by the authors as PB) with rGO has efficiently contributed to the adsorption of LiPSs. The S@PB material has been previously prepared by the coprecipitation of Na_2_Co­[Fe­(CN)_6_], and then by the physical adsorption of sulfur (Figure 10a). As the nanocubes present a rough surface, it indicates that probably most of the sulfur content is adhered to their surface. Furthermore, the SEM images of the S@PB@rGO indicated that the nanocubes are coated by a thin layer of rGO (Figure 10b). The S@PB material coated with rGO (S@PB@rGO) has shown superior performance to the S@PB as a cathode for Li–S batteries. The S@PB@rGO provided a capacity retention of ∼ 93.5% after 100 cycles at 0.5 C (reversible capacity of ∼ 858 mAh g^–1^), significantly higher than the capacity retention of ∼ 83.9% provided by the S@PB cathode without rGO, which showed a reversible capacity of ∼ 520 mAh g^–1^ at the same conditions (Figure 10d and Table 1). Furthermore, the S@PB@rGO cathode provided high-capacity retention to the system, and it showed high-capacity values under current densities up to 2C (Figure 10e). However, the S@PB@rGO provided a considerably lower resistance than S@PB, favoring the diffusion of Li^+^ in the sulfurized cathode. The reason for the superior performance of the S@PB@rGO was possibly due to the combination of the S@PBA composite with rGO, which contributed to the adsorption of LiPSs, as rGO presents active sites to provide the chemical adsorption of LiPSs, and its structure also permits physical confinement of LiPSs, as the authors stated^72^ (Figure 10c). Therefore, the procedure of coating sulfurized PBAs with a 2D carbon layer should be further considered when designing sulfur hosts that can effectively suppress the shuttle effect.

**Figure 10:**
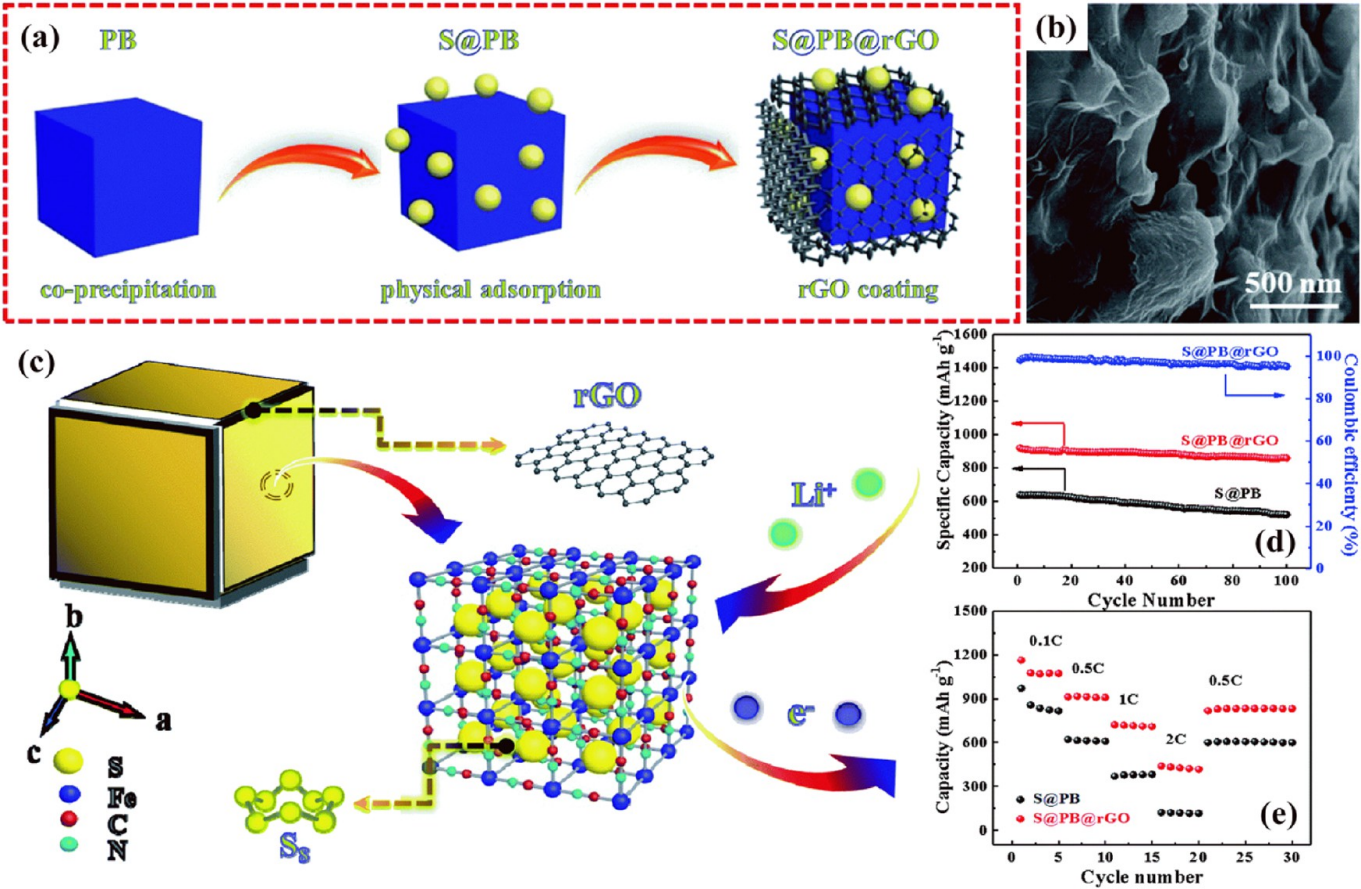
(a) Representation of the preparation procedure of the S@PB@rGO composite cathode. (b) SEM images of the S@PB@rGO composites nanocubes. (c) Diagram of the S@PB@rGO system during the discharge process. (d) Cyclability test and (e) rate capability for the cells comprising the S@PB and S@PB@rGO cathodes. Adapted with permission from ref 72. Copyright 2020, Royal Society of Chemistry.

## 5 PBA-Derived Materials for Lithium–Sulfur Batteries

Similar to many other coordination compounds, PBAs have also been strategic materials for the design of multifunctional-derived materials, covering a variety of derivatives with configurational entropy ranging from low to high.^104^ In fact, PBAs have been employed as self-sacrificial templates or precursors in the synthesis of a vast number of electrode materials, including alloys, metal oxides, metal hydroxides, metal phosphides, metal sulfides, and several others.^82^ Their attractiveness lies in the preservation of porous architectures and enhanced electrochemical performance following thermal treatment.^105^ In addition to their distinctive structural characteristics, PBA-derived electrocatalysts typically exhibit high specific surface areas and well-distributed active sites.^106^ Furthermore, PBAs can act as sources of both carbon and nitrogen, where nitrogen doping plays a key role in modulating the electronic structure of the catalytic surfaces, thereby improving their intrinsic activity.^106^

Interestingly, there has been a recent focus on designing numerous PBA-derived materials with high entropy, particularly due to the strong catalytic activity demonstrated by such high-entropy materials (HEMs). For instance, PBA-derived high-entropy alloys (HEAs) have been applied for efficient Fenton-like catalysis.^107^ indirect nitrite reduction to ammonia,^108^ etc. PBA-derived materials exhibit notable advantages over their PBA precursors, especially in electrochemical contexts. For example, pyrolysis of PBAs often results in structures with significantly improved electrical conductivity and thermal stability, making them well-suited as electrode materials. These transformations can result in a larger surface area and increased pore volume, which facilitates efficient ion and electron transport during electrochemical reactions. Additionally, PBA-derived materials allow for tunable morphology, porous structure, and multimetal compositions^109^ creating more active sites and adaptable electronic properties that enhance catalytic efficiency and improve redox kinetics relative to the original PBAs. Together, these features significantly boost performance and durability in energy devices, positioning PBA-derived materials as highly promising in energy storage and conversion, especially those high-entropy derivatives.

In this context, this section is devoted to the study of the various PBA derivatives presented in the literature and their application in Li–S batteries (Table 2). It discusses their properties to underscore emerging trends and critical insights within the field.

**Table 2:** Performance of PBA-Derived Materials Applied as Sulfur Hosts for Li–S Batteries

**Material**	**Material**	**Metals**	**Derivatization method**	**Sulfur content (wt %)**	**Sulfur loading (mg cm** ^**–2**^)	**Reversible Capacity (mAh g** ^**–1**^ **)/Capacity Retention (%)**	**Initial capacity (mAh g** ^**–1**^)	**WVW (V vs Li/Li+)**	**REF**
Oxide	Fe/CeO_2_–CNTs	FeCe	Thermal	80	1.1	1003/∼81% after 100 cycles @ 0.2C	1241.0 @ 0.2C	1.7 – 2.8	^90^
					4.2	412.0/∼54% after 50 cycles @ 0.2C	902.0 @ 0.2C		
	S/CoFe_2_O_4_@C	CoFe	Thermal	74.3	-	557/∼68.5% after 500 cycles @ 2C	816.0 @ 2C	1.7 – 2.8	^70^
	S/CoFe_2_O_4_			-	-	821/74% after 100 cycles @ 0.5C	753.0 @ 0.5C		
	Co_*x*_Fe_3‑x_O_4_@S	CoFe	Thermal	73	1	898.9/47% after 500 cycles @ 1 A/g	1301.6 @ 0.2 A/g	1.7 – 2.8	^84^
	Fe_2_O_3_@S	Fe		-		591.6/51 after 100 cycles @ 0.2 A/g	1221.7 @ 0.2 A/g		
	Co_3_O_4_@S	Co		-		531.1 47.8 after 100 cycles @ 0.2 A/g	1018.1 @ 0.2 A/g		
Sulfides	CuCo_2_S_4_–S	CuCo	Thermal	-	-	642/∼67% after 100 cycles @ 0.2 A/g	959.0 @ 0.2C	1.6 – 2.8	^89^
	MnCo_2_S_4_–S	MnCo		-	-	731/∼73% after 100 cycles @ 2 A/g	1002.0 @ 0.2C		
	NiCo_2_S_4_–S	NiCo		45	1.2	336/74% after 1000 cycles @ 1C	1283.0 @ 0.2C		
	AC-S	-		-	-	447/∼53% after 100 cycles @ 0.2C	960.0 @ 0.2C		
	rGO/S-CFS-_2_/CP		Liquid	-	∼1.5	629.9/55.9% after 400 cycles @ 0.2C	1126.5 @ 0.2C	1.7 – 2.8	^121^
	rGO/S-FeS_2_/CP	Fe		-		∼ 587.5/after 100 cycles @ 0.2C	1175.0 @ 0.2C		
	rGO/S-CP			-		-	1065.0 @ 0.2C		
	rGO/S			70		-	-		
	S@NiCo/NC	-	Thermal	-	1.2	∼980/∼17.5% after 1500 cycles @ 1C	1303.6 @ 0.1C	1.7 – 2.8	^88^
	S@NiCo–NiCoS/NC	-		-	1.2	∼800/1220 cycles @ 1C	1354.1 @ 0.1C		
	CoNi_2_S_4_	CoNi	Thermal	-	-	536.7/60% after 1000 cycles @ 1C	1231.8 @ 0.1C	1.7 – 2.8	^125^
Phosphides	Fe–Ni–P@NC	FeNi	Thermal	69	1.2	470.8/60% after 500 cycles @ 1C	806.4 @ 1C	1.7 – 2.8	^91^
	FCNP (S/10 wt %)	FeCoNi	Thermal	60	1.4–1.6	1091.6/69% after 100 cycles @ 0.2C	1091.6 @ 0.2C	1.7 – 2.8	^92^
	FCNP (S/20 wt %)					1172.3/75% after 100 cycles @ 0.2C	1172.3 @ 0.2C		
	FCNO (S/20 wt %)					854.5/52% after 100 cycles @ 0.2C	854.5 @ 0.2C		
	FCNO (S/10 wt %)					1051/100 cycles @ 0.2C	1051 @ 0.2C		
	CoSP/rGO/S	Co	Thermal	73	1.2–1.5	400.0/58.6% after 900 cycles @ 1C	670.0 @ 1C	1.7 – 2.8	^85^
	CNT/NFP/PP	NiFeP	Thermal	-	-	681.4/79.9% after 300 cycles @ 1C	1244 @ 0.2C	1.7–2.7	^126^
Alloys	HEMNS@GR	VCrMoNbZr	Liquid		1.4	404/∼70% after 500 cycles @ 0.1C	1193 @ 0.1C	1.7–2.6	^127^
	S@CNCF-3–800		Thermal	73.56	1.2–1.8	802/∼26.1% after 100 cycles @ 0.5C	1315 @ 0.2 C	1.7–2.7	^87^
	CoFe@C/S	CoFe	Thermal	59	-	568/∼56.7% after 100 cycles @ 0.1C	1105.3 @ 0.1 C	1.7 – 2.8	^83^
	S/CoFe@NC/PPC	CoFe	Thermal	-	-	447.4/48.9% after 500 cycles @ 1C	915.6 @ 1C	1.7–2.8	^112^
Metal NP’s	S/Co-NPC-MCs	Co	Thermal	79.6	1.9	941.0/68% after 200 cycles @ 0.5C	1192.0 @ 0.5C	1.7–2.8	^81^
MXenes	MXene@PBA	MnFeTi	Liquid	-	1	583.0/69% after 500 cycles @ 0.5C	1220 @ 0.1C	1.5–2.8	^128^
MOF	Fe_3_C-NCNT	FeCoNi	Thermal		4	490/44% after 200 cycles @ 2C	1456 @ 0.2C	1.6–2.8	^129^
Others	FeCoPS_3_/NC	CoFe	Thermal		2	595.3/63% after 1000 cycles @ 1C	1244.4 @ 0.1C	1.7–2.8	^130^
	Fe/Fe_3_C/FeN_0.0324_	Fe	Thermal	-	4.5	748/73% after 300 cycles @ 0.5C	1025 @ 0.5C	1.7–2.8	^131^

### 5.1 PBA-Derived Alloys

Alloy catalysts have been considered highly promising host materials for enabling fast kinetics in Li–S batteries, primarily due to their intrinsic high conductivity, which effectively minimizes interface resistance on the cathode.^8^ In this context, multielemental catalysts offer a strategic advantage over conventional single-metal catalysts, which cannot effectively accelerate the stepwise sulfur redox reactions involving 16-electron transfers and various Li_2_S_n_ (*n* = 2–8) intermediates.^110^ In fact, Gonçalves et al.^8^ reported that within the broad range of FeNiCo-containing medium- and high-entropy materials (ME- and HEMs), alloy-based catalysts stand out as exceptionally promising. Key advantages of medium- and high-entropy alloys (ME- and HEAs) include their high conductivity, improved electrochemical performance, enhanced chemisorption affinity, and notable progress in synthesis methodologies.^8^ Besides, incorporating multiple elements within HEAs significantly increases the number of catalytically active sites, which not only boosts rate capability but also improves the cycling stability of assembled devices.^110^ The strategic design of alloy composition, ranging from low- to high-entropy, can introduce essential synergistic effects in Li–S battery catalysis. For example, experimental and theoretical studies indicate that Mn and Mo are pivotal in the polysulfide reduction process within CoNiCuMnMo-containing HEAs, while Ni and Co significantly contribute to sulfide oxidation. Meanwhile, Cu plays a regulatory role in the redox reactions, enabling the HEA to demonstrate exceptional bidirectional catalytic performance.^111^

The advances highlighted above clearly demonstrate that metal nanoparticles (NPs) and/or alloys present great prospects as host catalysts for Li–S batteries, especially when designed with suitable sizes, morphologies, and compositions. In this context, the derivatization of PBAs has also led to the development of new NPs or alloys with optimized properties, but currently focuses on the design of low-entropy alloys (LEAs).

Interestingly, the main results achieved are due to the synergistic interaction between PBA-derived NPs or alloys and carbon-based materials, which exhibit catalytic effects that enhance electrochemical performance. In fact, significant progress has been reported in the development of CoFe-containing binary alloys with carbon material derived from organic compounds (melamine,^87^ polydopamine,^83^ etc.) or biomass.^112^

In a related study, Jing et al.^112^ synthesized a composite material consisting of biochar-coated alloy NPs derived from PBA and pomelo peel biomass as precursors. The synthesis involved depositing CoFe-PBA onto pomelo peels in solution, followed by high-temperature calcination. During calcination, the pomelo peel (PP) biomass was converted into N-doped porous carbon (NC), while Co and Fe ions were reduced to form CoFe-containing LEA NPs (referred to as CoFe@NC/PPC). This N-doped porous carbon matrix not only offers excellent electrical conductivity but also binds sulfur effectively. Additionally, the CoFe NPs enhance sulfur adsorption. The N-rich porous structure and CoFe alloy effectively mitigate the shuttle effect, while the biochar matrix improves the conductivity of the sulfur cathode, facilitating complete sulfur utilization, as can be observed in Figure 11a. Figure 11b–c shown the TEM images of S/CoFe@NC/PPC, where the CoFe NPs can be observed in the carbon matrix. As a result, the initial specific capacity reached approximately 915.6 mAh g^–1^, retaining 447.4 mAh g^– 1^ after 500 cycles at 1C (capacity retention of ∼ 48.9%).^112^

**Figure 11:**
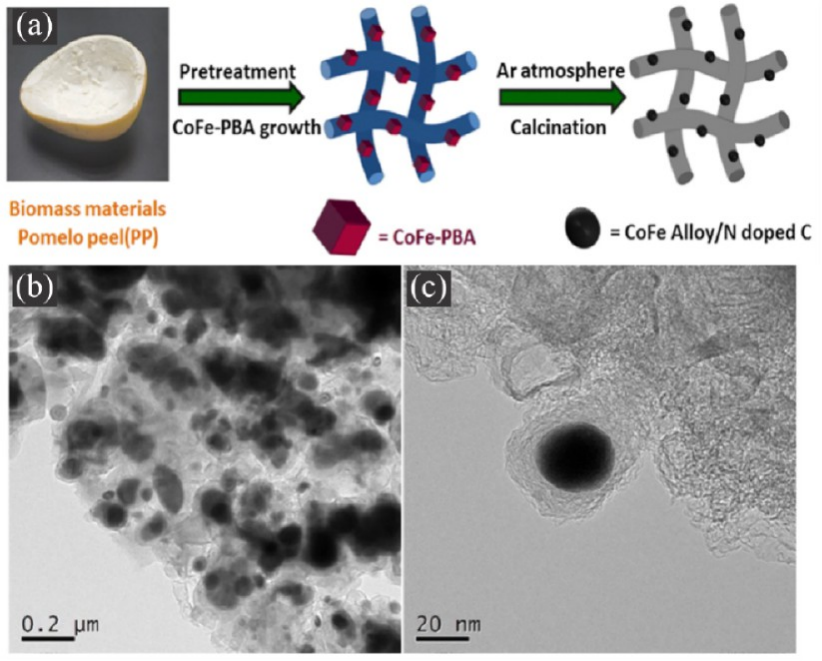
(a) The schematic representation of the CoFe@NC/PPC synthesis procedure. (b-c) TEM images of S/CoFe@NC/PPC. Reproduced with permission from ref 112. Copyright 2019, Springer-Verlag GmbH Germany, part of Springer Nature.

Even more promising results have been achieved using melamine as a carbon source, largely due to its high nitrogen content, which makes it ideal for creating N-doped carbon materials.^87^ CoFe/N-doped mesoporous carbon hybrids were synthesized via straightforward pyrolysis of CoFe-PBA and melamine, with careful control of the PBA-to-melamine weight ratio and the annealing temperature to achieve a tailored structure.^87^ The composite exhibits exceptional electrochemical performance, including a high initial discharge capacity of 1315 mAh g^–1^ at 0.2C, great rate capability of 724 and 496 mAh g^–1^ at 2 and 5C rates, respectively, and outstanding cycling stability (528 and 367 mAh g^–1^ at 2 and 5C after 500 cycles, respectively). The synergistic effect of the mesoporous carbon matrix, uniformly sized CoFe alloy particles, and nitrogen heteroatoms effectively confines sulfur species, with meso- and micropores physically trapping the sulfur. Additionally, CoFe-N_*x*_ moieties enhance the composite’s electronic conductivity and provide active sites for the chemical absorption and catalytic conversion of polysulfides, effectively mitigating the shuttle effect. The mesoporous structure also buffers volume changes during cycling. This strategy introduces a promising approach for designing N-doped mesoporous carbon matrices with embedded CoFe nanoparticles for high-performance Li–S battery cathodes, providing high power density and large capacity.

### 5.2 PBA-Derived Metal Oxides

Despite the widespread design and use of metal oxides derived from PBAs for various energy applications, there has yet to be a significant prevalence of these PBA derivatives as catalysts in Li–S batteries. As expected for a relatively simple standalone thermal process,^82^ a wide range of low- to high-entropy oxides (LEOs/MEOs/HEOs), as well as mixtures of various metal oxides, have been reported in recent years.^113,114^ Meanwhile, only a limited number of unary and binary compositions have been developed as host materials for the conversion catalysis of LiPSs. On the other hand, these early advancements highlight significant future opportunities for designing ME- and HEOs derived from ME- and HE-PBAs, respectively.

Interestingly, PBA-based precursors can be designed to achieve either a single phase (especially necessary for the development of MEMs and HEMs) or multiple phases. Variations in oxygen affinity of the metal species of the PBA can result in mixtures of single-metal oxides or mixed-metal oxides.^82^ For instance, even under an inert nitrogen atmosphere, CeO_2_ was obtained by annealing a binary FeCe-PBA precursor at 900 °C, as expected due to the strong affinity of Ce ions for oxygen.^90^ On the other hand, Fe ions form catalytic Fe particles under the same reaction conditions.^90^ In this context, the design of single-phase PBA-derived materials depends not only on the synthesis conditions but also on the composition of the precursor. Elements with high oxygen affinity tend to form segregated phases more easily. Therefore, incorporating such elements can be particularly challenging in the development of HEMs and even more so in low-entropy systems where these elements are present in significant concentration.

On the other hand, the design of binary low-entropy oxides (LEOs, single-phase mixed-metal oxides) has been successfully achieved using PBA precursors, mainly those containing elements from the first transition series. In one such study, Gu et al.^70^ developed CoFe_2_O_4_ nanocages enveloped in a thin carbon layer (CoFe_2_O_4_@C) as a highly efficient sulfur host for Li–S batteries. As summarized in Figure 12a, the derivatization of CoFe-PBA occurs through annealing at 400 °C for 2 h in the air, resulting in the formation of a hollow CoFe_2_O_4_ nanocage. The as-synthesized CoFe_2_O_4_ was then coated with a ∼ 7 nm carbon layer by carbonizing polydopamine (PDA) through annealing in argon at 600 °C. For application, sulfur was infiltrated into the hollow CoFe_2_O_4_@C by a melt-diffusion process at 155 °C, forming the S/CoFe_2_O_4_@C composite. Figure 12b–f confirms the cubic shape and elemental composition of the S/CoFe_2_O_4_@C from SEM, TEM, HRTEM, and EDS analysis. Similar to what was reported for pristine PBAs, the carbon layer plays a crucial role in enhancing the electrical conductivity of the PBA-derived oxide (2.38 S cm^–1^ for CoFe_2_O_4_@C and 4.24 × 10^–5^ S cm^–1^ for CoFe_2_O_4_), which is fundamental to achieving a promising material for battery electrodes. This strategy leads to an enhanced electron transfer process and kinetic performance. The electrochemical performance of the S/CoFe_2_O_4_@C exhibited significantly greater stability during 500 cycles at a current density of 2C and good rate performance compared to its counterpart without carbon-coating, which can be considered a promising metric for Li–S batteries.

**Figure 12:**
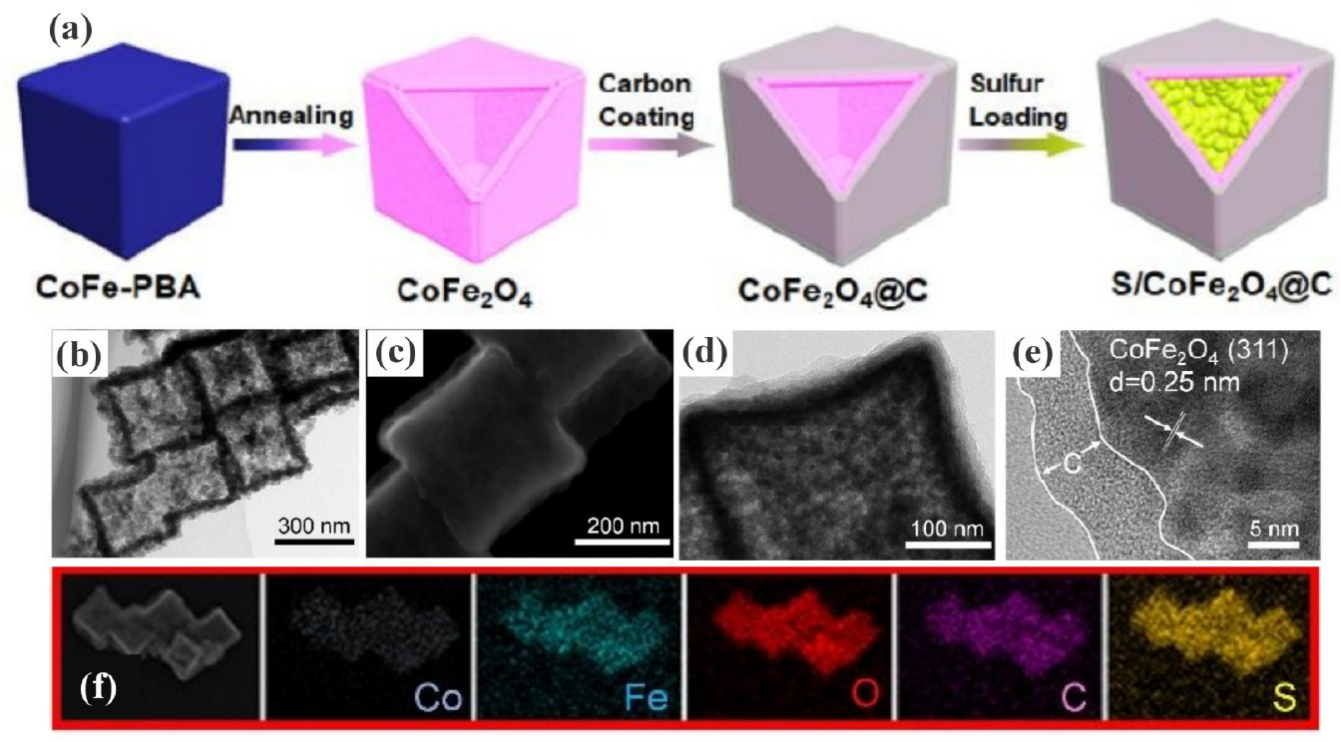
a) Schematic illustration of the formation of the S/CoFe_2_O_4_@C composite. b) TEM image of CoFe_2_O_4_. c) SEM image, d) TEM image, and e) HRTEM image of CoFe_2_O_4_@C. f) Corresponding element mappings of the S/CoFe_2_O_4_@C composite. Reproduced with permission from ref. 70. Copyright 2020, Royal Society of Chemistry.

It is important to emphasize that, even though PBA precursors contain elements from the first transition series, the entire synthesis process must be carefully considered to obtain metal oxide derivatives with either a single phase or multiple phases. In other words, the experimental procedure must be thoughtfully selected, taking into account the target derivative and, in particular, the composition of the pristine PBA. For instance, even using a low-entropy CoFe-PBA similar to the one previously reported by Gu et al.^70^ Chen and colleagues^84^ obtained relatively different results, particularly characterized by the segregation of the unary PBA-derived metal oxides of both elements. In this study, these differences can be primarily attributed to the addition of a pretreatment step, specifically the etching of the PBA precursor, which is commonly performed to create porosity and hollowness^82^ as summarized in Figure 4. During the etching process, the etchant targets the most reactive or structurally weak areas, typically the corners or edges, resulting in the formation of cages.^82^ The hollow nanocages derived from PBA-based heterostructures, featuring a highly interconnected pore architecture, not only prevent LiPS diffusion by forming metal–sulfur bonds but also enhance LiPS conversion kinetics. According to the authors, the hollow porous structure physically confines LiPSs and accommodates volume changes, significantly improving the rate capability and cycling stability of the hollow composite-based electrode. As a result, the electrode based on hollow metal oxide demonstrates an impressive initial capacity of 1301.6 mAh g^–1^ at a current density of 200 mA g^–1^, with great stability during the rate capability experiment. However, cycle stability at high current density can still be considered a challenge, since capacity retention is less than 50% after 500 cycles.

Despite the promising results, the design of PBA-derived metal oxides remains in its early stages. To date, medium- and high-entropy oxides (MEOs/HEOs) derived from PBAs for Li–S battery applications have not been reported in depth. In fact, although Du et al.^17^ reported a series of PBA-derived metal oxides (Figure 13a–i), including CoNiFe-oxide, NiCuFe-oxide, NiZnFe-oxide, CuZnFe-oxide, CoMnFe-oxide, and CoNiCuMnZnFe-oxide, further research is needed to optimize their application in Li–S batteries, particularly in the context of MEOs and HEOs.^17^ Even greater challenges are anticipated in this area, given the varying oxygen affinities of the different metals involved. However, to successfully derive metal oxides or mixed-metal oxides from PBAs, particularly for MEOs and HEOs, the temperature and duration of the treatment must be precisely controlled. Besides, in the case of MEOs and HEOs, rapid synthesis methods have also been employed to prevent phase segregation. Metal oxide derivatization can be achieved across a wide temperature range, from 250 to 900 °C, depending on the desired oxide phase. In addition, pre- and post-treatments can be used favorably.^82^

**Figure 13:**
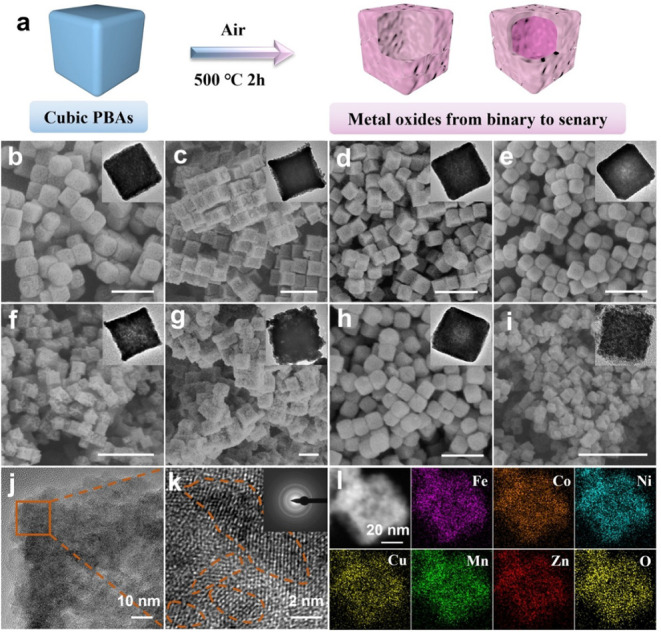
(a) The synthesis process for metal oxides. SEM and TEM images for (b) NiFe-oxide, (c) CoFe-oxide, (d) CoNiFe-oxide, (e) NiCuFe-oxide, (f) NiZnFe-oxide, (g) CuZnFe-oxide, (h) CoMnFe-oxide, and (i) CoNiCuMnZnFe-oxide. The SEM images have scale bars set to 500 nm. (j,k) High-resolution TEM images and selected area electron diffraction (SAED) pattern of CoNiCuMnZnFe-oxide. (l) High-angle annular dark-field STEM image and associated elemental mapping of CoNiCuMnZnFe-oxide. Reproduced with permission from ref. 17. Copyright 2022, Wiley-VCH GmbH.

### 5.3 PBA-Derived Metal Sulfides

In recent years, significant efforts have focused on designing metal sulfides for various electrocatalytic and energy storage applications.^1,115,116^ These promising studies highlight the advantageous properties of metal sulfides in electrochemical applications, particularly when compared to their metal oxide counterparts.^1^ In fact, replacing oxygen atoms with sulfur atoms can further enhance the electrochemical properties and broaden the potential applications of these materials. Sulfide, being a softer ligand than oxide, offers richer redox activity, greater stability, higher charge/electron transfer rates, more adaptable structural characteristics, and lower band gap energy due to synergistic effects from the various active sites with multiple oxidation states (especially in the case of binary and ternary sulfides, or in high-entropy sulfides – HESs.^117^ Moreover, substituting oxygen with sulfur creates more flexible systems due to the lower electronegativity of sulfur, which minimizes volume changes during charge and discharge cycles, leading to structurally more stable materials. The reduced band gap also boosts conductivity, enabling faster electron transport throughout the electrode material.^116^

On the other hand, although the concepts of ME- and HEMs are already being applied to the design of electrocatalysts^118^ and energy storage materials. based on PBA-derived metal sulfides, their use remains relatively infrequent in the literature. However, some low-entropy compositions have been reported, mainly binary sulfides. In analogy to metal oxides, the main low-entropy sulfides (LESs) are also based on Co-containing spinels. Co-based sulfides have been shown to not only adsorb soluble polysulfides but also to enhance the redox kinetics of these species, thanks to their excellent conductivity.^89^ Moreover, among LESs, binary metal sulfides like NiCo_2_S_4_ have found extensive application in electrocatalysis and energy storage due to their superior electrical conductivity and abundance of active sites compared to single-metal sulfides.^89^

Interestingly, the promising electrochemical performance of NiCo_2_S_4_-based LES catalysts was supported by Li et al.^89^ Compared with sulfur-rich activated carbon (AC-S) cathodes and other binary metal sulfides (MnCo_2_S_4_–S and CuCo_2_S_4_–S) with equivalent sulfur content and loading mass, the NiCo_2_S_4_–S exhibited the best performance. For instance, the NiCo_2_S_4_–S cathode demonstrates a high initial discharge capacity of 1283 mAh g^–1^, retaining 787 mAh g^–1^ after 100 cycles (capacity retention of ∼ 61.3% at 0.2C). In comparison, the initial specific capacities for the MnCo_2_S_4_–S and CuCo_2_S_4_–S composite cathodes are 1002 mAh g^–1^ and 959 mAh g^–1^, respectively, indicating that even these binary metal sulfide composites fall short of the performance achieved by the NiCo_2_S_4_–S composite cathode. The PBA-derived binary sulfide showed a long-term cycling performance with a capacity decay of 0.026% per cycle after 1000 cycles at a rate of 1C (capacity retention of ∼ 74%), which can be understood as great progress for an electrode with a high sulfur loading of 3 mg cm^–2^.^89^ The authors suggest that the polar NiCo_2_S_4_ host, with its excellent electron conductivity, effectively confines polysulfides and accelerates their redox reactions.

Still on NiCo-containing catalysts derived from NiCo-PBA, Zhu and colleagues^88^ highlighted the synergistic effects of combining a binary alloy and binary sulfide with N-doped carbon, achieving exceptional rate performance and extended lifespan in Li–S devices. In more detail, a heterostructure comprising a NiCo alloy and mixed NiCo-based sulfides (NiCo_2_S_4_ and Co_9_S_8_, referred to as NiCoS), integrated within an NC matrix (labeled NiCo–NiCoS/NC), was synthesized through a simple pyrolysis process followed by controlled vulcanization of NiCo-PBA. The NiCo–NiCoS/NC structure imparts the scaffold with enhanced conductivity and good sulfur affinity. At the same time, the NC matrix forms a robust network that ensures the effective dispersion of active nanoparticles. As a result, the NiCo–NiCoS/NC-based cell achieves a high discharge capacity of 1345.1 mAh g^–1^ at 0.1C, an impressive rate capability of 692.1 mAh g^–1^ at 5 C, and a lifespan exceeding 1500 cycles, with a capacity fading of 0.055% per cycle at 1C (capacity retention of ∼ 17.5%). Despite the low-capacity retention reported for the NiCo–NiCoS/NC structure, alloy-containing electrodes, particularly those with medium and high entropy, are recognized for their high catalytic activity in favoring the conversion of LiPSs.^8^ On the other hand, previous studies indicate that the NC matrix can significantly enhance the performance of Li–S batteries.^119,120^ Recent studies suggest that N-doping enhances the nucleation rate of Li_2_S. This is further supported by density functional theory (DFT) analysis, which shows that N-doped substrates exhibit higher binding energies for Li_2_S compared to carbon-only substrates.^120^

Beyond the metal composition and incorporation of carbon-based materials, another approach to enhance catalytic kinetics in LESs is by tuning the concentration of the PBA precursor. In this context, Huang et al.^121^ identified a correlation between the polysulfide adsorption capability and the metal ratio within the precursor. A series of Co–Fe sulfides with varying Co-to-Fe metal atom ratios was synthesized through a simple solvothermal method. The ratios were precisely controlled by adjusting the molar ratios of CoCl_2_·6H_2_O to FeCl_2_·6H_2_O to 0:1, 1:1, 2:1, 5:1, 8:1, and 10:1. The composite interlayer with 2:1 ration (denoted as CFS-2/CP) enhances LiPSs conversion through a dual mechanism: the porous architecture of the carbon paper acts as a physical barrier, restricting LiPSs diffusion, while binary Co–Fe sulfides provide abundant catalytic and chemical binding sites. The synergy between Co and Fe ions adjusts electronic structure and binding affinity, promoting sustained catalytic conversion throughout cycling. This interlayer structure accelerates electron and ion transport, resulting in improved cycling stability. Consequently, Li–S batteries with the CFS-2/CP interlayer exhibit an initial capacity of 1126.5 mAh g^–1^ and a minimal capacity loss of 0.11% per cycle over 400 cycles (capacity retention of ∼ 56%), demonstrating the promise of binary sulfide-based designs for enhanced battery performance.

### 5.4 PBA-Derived Metal Phosphides

Metal phosphides have attracted considerable attention for electrocatalytic applications due to their superior electrical conductivity and remarkable catalytic properties compared to metal oxides and metal sulfides.^122^ Transition metal phosphides, in particular, stand out as promising electrocatalysts because of their unique crystal structure featuring triangular prismatic geometry and a high density of unsaturated surface atoms. This structure imparts strong metallic character and excellent conductivity, positioning them as highly effective catalysts.^122,123^

Recently, metal phosphides have been extensively explored as promising electrocatalysts for Li–S battery devices. Unlike other metal compounds, phosphides can be synthesized through relatively simple and mild processes. In this context, several recent review articles have been published to underscore these emerging trends.^122–124^

Current trends also reveal the use of coordination compounds in preparing metal phosphides, especially PBA derivatives. Interestingly, despite using binary PBA or ternary PBA-based precursors, all reported PBA-derived metal-phosphides exhibit multiphase compositions (and polycrystalline nature) of unary metal phosphides. In fact,no medium-entropy (MEPs) or high-entropy phosphides (HEPs) derived from PBAs have been prepared to date. For instance, employing the composite formation strategy, Song et al.^91^ designed a Fe–Ni–P@nitrogen-doped carbon (Fe–Ni–P@NC) derived from FeNi-PBA, as shown in Figure 14a–g. The polycrystalline nature and high crystallinity of Ni_2_P and Fe_2_P nanocrystals were verified through selected area electron diffraction (SAED, Figure 14h) and X-ray diffraction (XRD) analysis. Despite phase segregation, Fe–Ni–P@NC composites enhance the adsorption of LiPSs.^91^ Besides, nitrogen-doped carbon derived from the CN^–^ groups in PBAs boosts electrode conductivity in Li–S batteries, which can be confirmed by DFT calculations. Consequently, Li–S batteries incorporating S@Fe–Ni–P@NC composites as the cathode demonstrated improved rate performance compared to the S@Fe–Ni@NC (Figure 14i), and exceptional cycling stability, delivering a discharge capacity of 470.8 mAh g^–1^ after 500 cycles at 1C, as can be observed in (Figure 14j). This performance notably surpasses that of S@Fe–Ni@NC cathodes (synthesized under identical experimental conditions but without the addition of NaH_2_PO_2_·H_2_O), which exhibited a significantly lower capacity, declining to 268.5 mAh g^–1^ after 500 cycles.^91^

**Figure 14:**
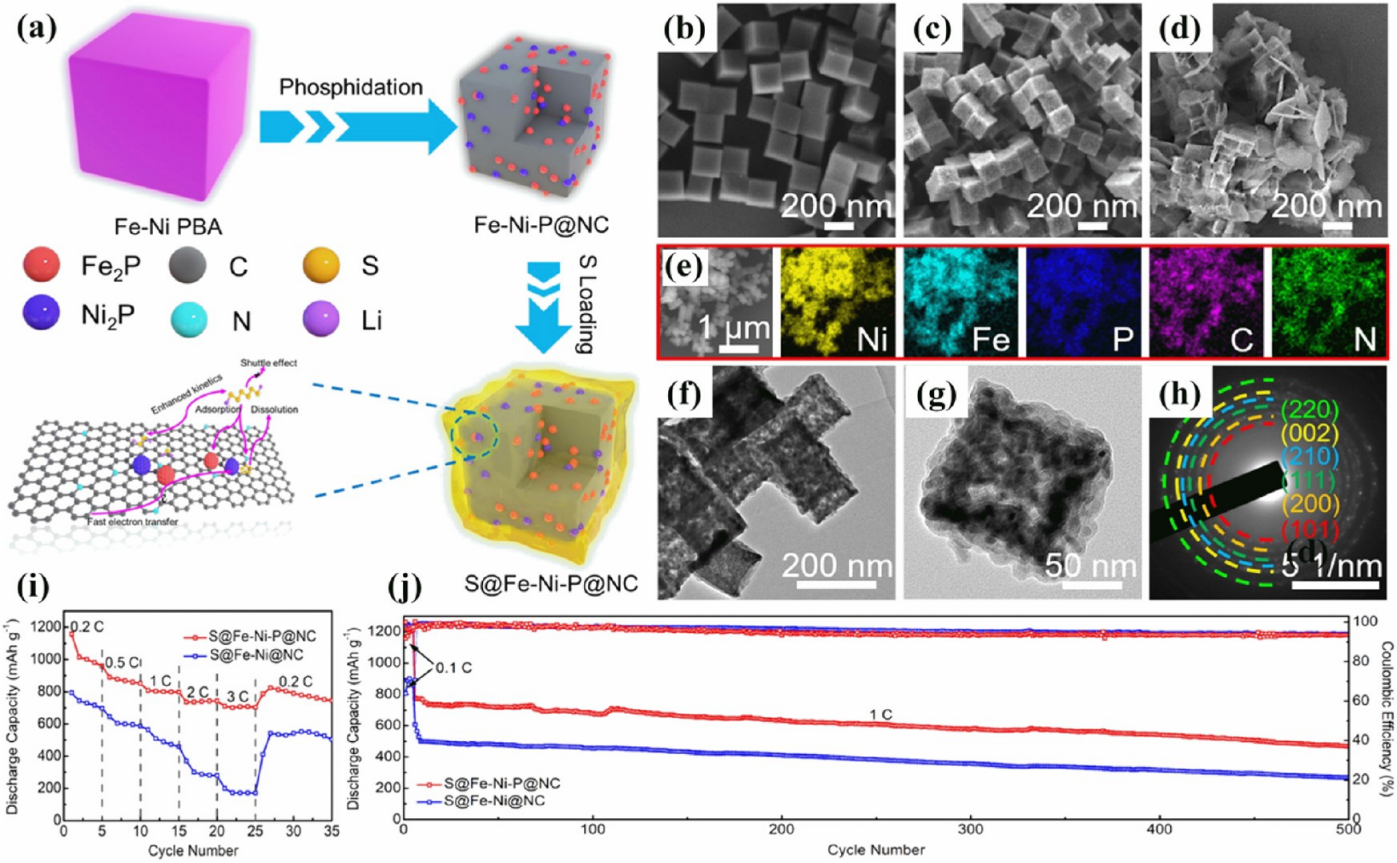
(a) Schematic diagram of the preparation of S@Fe–Ni–P@NC composites. SEM micrographs of (b) Fe–Ni PBA, (c) Fe–Ni–P@NC, and (d) Fe–Ni@NC. (e) Elemental distribution map of the Fe–Ni–P@NC sample. (f, g) TEM micrographs illustrating the morphology of Fe–Ni–P@NC. (h) SAED pattern corresponding to Fe–Ni–P@NC. (j) Rate performance from 0.2C to 3C, and (g) cycling performance at 1C of the Li–S batteries with S@Fe–Ni–P@NC and S@Fe–Ni@NC electrodes. Reproduced with permission from ref. 91. Copyright 2021, Elsevier Inc. All rights reserved.

Similar results were reported in the work of Yang and collaborators^92^ since the use of a ternary FeCoNi-PBA was employed as a precursor of a composite containing a mix of unary FeP, CoP, and Ni_2_P (named FCNP).^92^ The FCNP outperforms FCNO (combination of Fe_3_O_4_, CoO, and NiO) as a catalyst in Li–S battery systems due to its moderate LiPS adsorption capability, high conductivity, and shorter pathway for LiPS evolution, enhancing electrocatalytic efficiency.^92^ While FCNO exhibits slightly stronger LiPS affinity, as confirmed by UV–vis and DFT calculations, its poor conductivity limits LiPS diffusion and reaction to surface interactions, which constrains the catalytic role of the Fe–Co–Ni centers. In contrast, FCNP enables the entire adsorption, diffusion, and conversion of LiPS directly on its conductive surface, thereby optimizing the cooperative effect of trimetallic centers. Furthermore, DFT results indicate that the phosphide components (FeP, Ni_2_P, CoP) in FCNP exhibit varied adsorption energies, with CoP having a smaller band gap and a stronger catalytic role in LiPS conversion. This combination of moderate adsorption, high conductivity, and catalytic synergy between the metal phosphide centers grants FCNP-superior electrocatalytic performance and stability for Li–S batteries. Cathodes with 20 wt % FCNP exhibit a higher initial capacity (1172.3 mAh g^–1^) and a low decay rate (0.25% per cycle at 0.2C), maintaining structural integrity over 100 cycles. Additionally, the composition of FCNP offers lower polarization voltages and a smaller charge transfer resistance, allowing efficient sulfur redox reactions and a more stable cycle life. In contrast, the lower conductivity and weaker cycle performance of FCNO (decay of 0.48% per cycle at 0.2C) limit its suitability for long-term battery applications.^92^

Although PBA-derived metal phosphides offer advantages over many analogous materials (metal oxides, metal sulfides, etc.), these highlighted works reveal potential challenges in designing medium- and high-entropy phosphides (ME- and HEPs) derived from PBAs. Achieving a single-phase structure is critical. The formation of multiple phases can undermine the expected synergistic effects, reducing conductivity, catalytic efficiency, and structural stability. Consequently, careful control over synthesis conditions and compositional design is essential to fully realize the benefits of ME- and HEPs in energy storage applications like Li–S batteries.

## 6 Conclusions and Outlook

Prussian Blue (PB) and its analogues (PBAs) are coordination compounds that have attracted increasing attention in strategic areas such as energy conversion and storage. More recently, significant advances have been reported in the design of PB- and PBA-based catalysts for Li–S batteries, particularly due to their synergistic effects in promoting the conversion of lithium polysulfides (LiPSs) and enhancing overall electrochemical performance. Indeed, PBAs have emerged as promising sulfur host materials due to their abundance of active sites capable of chemically adsorbing LiPSs and effectively mitigating the shuttle effect. This is particularly true for multimetal-containing porous coordination polymers, which not only provide strong chemical interactions but also enable the physical confinement of LiPSs within their porous frameworks.

Broadly, recent studies have shown that ternary PBAs such as FeCoNi-PBA (the most reported composition) outperform unary and binary counterparts as sulfur hosts in Li–S batteries due to enhanced chemisorption and synergistic effects. Interestingly, coating PBAs with conductive polymers (e.g., polypyrrole—PPy) has been reported as a promising strategy that improves LiPS confinement and ion transport, increasing capacity retention. For instance, PPy accelerated LiPS conversion via thiosulfate formation, reducing shuttle effects. Other polymers like PEDOT have similarly enhanced capacity and rate capability. These results evidence the effectiveness of the polymer-coating strategy in enhancing both the capacity and power performance of sulfur-based electrodes, making it a promising approach for high-power Li–S battery applications. On the other hand, high-entropy PBAs (HE-PBAs) with five metal elements also show promise as advanced sulfur hosts. Besides, combining PBAs with carbon-based materials, such as rGO, enhances the adsorption of LiPSs by providing both physical confinement and chemical adsorption. These findings suggest that combining multimetal PBAs with conductive polymers or carbon-based materials is a promising strategy for improving Li–S battery performance and should be explored further, especially considering the emerging nature of research on HE-PBAs.

Despite the promising progress achieved with PBAs and their higher-entropy counterparts, these materials still fall short of fully meeting the criteria for advanced Li–S battery electrodes. In practical applications, the performance of PBA-based and high-entropy analogues largely hinges on enhancing their electrical conductivity and optimizing the configuration of redox-active sites, critical aspects that must be addressed to advance their effectiveness as catalytic electrode materials Li–S batteries. From this perspective, transforming PBAs into alloys, oxides, sulfides, and phosphides from low- to high-entropy design and/or the preparation of carbon-based composites has been highlighted as a promising strategy that enables enhanced conductivity, catalytic activity, and structural stability, crucial for overcoming challenges like polysulfide shuttling and sluggish redox kinetics. For instance, PBA-derived metal sulfides have demonstrated outstanding potential for Li–S battery applications due to their enhanced redox activity, structural stability, and superior conductivity compared to metal oxide counterparts. Among them, low-entropy sulfides such as NiCo_2_S_4_ stand out for their remarkable cycling performance and polysulfide confinement capabilities. Despite the promising outcomes, studies involving medium- and high-entropy sulfides remain scarce, representing an open avenue for future research. Besides, while their synthesis from PBA precursors offers a versatile and straightforward route, all reported PBA-derived metal phosphides thus far exhibit multiphase, polycrystalline compositions limited to unary systems, with no successful demonstration of medium- or high-entropy phosphides to date for Li–S technologies. These findings suggest a significant potential for further exploration of entropy-engineered PBA-derived materials with strong synergistic effects and rich active sites in advanced Li–S batteries.

Still, for PBA-derived materials as catalysts for advanced Li–S batteries, the literature reports progress in integrating PBA-derived nanoparticles, metal oxides, metal sulfides, or metal phosphides with carbonaceous matrices, particularly N-doped porous carbon (NC), CNT, and rGO, leading to composites with superior sulfur confinement, improved electron/ion transport, and tailored porosity. For instance, the synergy between the N-doped mesoporous carbon matrix and alloy ensures both physical confinement and strong chemical anchoring of sulfur species. In fact, among the PBA-derived materials reviewed, carbon composites containing metal alloys stand out as some of the most promising candidates in terms of catalytic activity. Such architecture enables the development of Li–S batteries with high power density and large capacity, maintaining excellent performance at elevated charge rates of 2C and 5C. Strategies incorporating binary or ternary sulfide compositions, heterostructures with N-doped carbon, and compositional tuning of PBA precursors have further improved electrochemical properties, including rate capability and cycling stability.

Although extensive research has been dedicated in recent years to developing PBA and PBA-derived electrode materials by leveraging their compositional and structural versatility, several key challenges and limitations remain to be addressed before their practical implementation in next-generation energy storage systems. In particular, the application of PBA-based materials in Li–S batteries is still at an early developmental stage. Therefore, to tackle the existing obstacles and unlock their full potential, future research directions and strategic perspectives are outlined and summarized in Scheme 2, as highlighted below:

1) The synthesis of ME- and HE-PBAs still requires further investigation, particularly regarding the design of PBAs with reduced dimensions, as the high surface area is a key factor in catalytic performance. Moreover, given the increasing demand for batteries in the context of the global energy transition, the development of highly scalable synthesis routes is urgently needed. In this regard, spray-drying techniques and microwave-assisted synthesis emerge as promising strategies, offering the potential for continuous, large-scale production while also enabling better control over particle size and crystallinity. On the other hand, beyond the large-scale production and particle size control of PBAs and their derivatives, the catalyst composition must also be carefully optimized from an economic perspective to enable practical applications. Therefore, cobalt-free formulations and the exclusion of noble metals should be prioritized in future material designs. In fact, recent studies^132,133^ reinforce the concern over cobalt availability, especially under high-demand growth scenarios that may lead to supply shortages as early as 2028. Nickel, though less critical in the short term, may also face constraints over the long run. These results indicate that PBA-based materials, particularly those with optimized compositions, could offer a more cost-effective and scalable option, provided that strategies such as cobalt recycling and diversification of supply sources are adopted. Moreover, Fe-, Mn-, and Cu-rich compositions should be further investigated for their potential economic advantages, along with the use of Co and Ni as dopant metals to enhance performance. This perspective strengthens the case for their use in future Li–S battery technologies.
2) Considering recent reports highlighting the formation of multiphase PBA-derived materials (particularly in the case of PBA-derived metal phosphides), the development of strategies to obtain single-phase compounds is essential for advancing high-entropy materials (HEMs) derived from PBAs. In this context, the use of ultrafast derivatization methods emerges as a promising pathway. Among the most promising techniques that can be explored for designing PBA-derived materials are flash Joule heating, spray pyrolysis, microwave-assisted synthesis, and the sol–gel combustion process, all of which are capable of promoting the rapid and controlled formation of single-phase products.
3) The fundamental experiments and theoretical understanding of catalytic reactions involved in polysulfide conversion on high-entropy materials are still at an early stage. To enable faster and more impactful progress, the use of advanced *in situ* and *operando* characterization techniques is essential, as they provide real-time insights into reaction intermediates and mechanisms.^134^ Among these, advanced spectroscopic methods utilizing synchrotron radiation play a pivotal role in uncovering the true catalytic pathways and guiding the rational design of optimized electrode materials for Li–S batteries.
4) Due to the intrinsic complexity of medium- and high-entropy systems, there remains substantial scope for theoretical studies aimed at elucidating the fundamental properties and underlying mechanisms of polysulfide conversion in ME- and HE-PBAs, as well as their derived materials. Computational approaches such as density functional theory (DFT), molecular dynamics (MD), and machine learning-assisted simulations can offer valuable insights into electronic structures, ion transport pathways, and catalytic behaviors. Besides, for the PBA-derived materials, the combination of predictive modeling with high-throughput simulations and machine learning makes it possible to navigate the vast compositional and structural space of these materials more efficiently, ultimately accelerating the development of high-performance cathodes for Li–S battery technologies. These tools are essential to guide the rational design of high-performance ME- and HE-cathode materials for next-generation Li–S batteries.
5) As previously discussed, misconceptions are often encountered and perpetuated in the literature, particularly when the concept of high entropy is applied to materials containing multiple metal sites, such as PBAs and many others (e.g., spinel oxides and perovskites). To prevent such misunderstandings, it is crucial to calculate the configurational entropy accurately, as not all multimetal PBAs with five metal ions in total, or five occupying the N-coordinated site, qualify as HE-PBAs. This careful evaluation should also extend to LE- and ME-PBAs, where configurational entropy calculations should serve as a simple foundational criterion to ensure proper classification. In this context, elemental quantification of PBAs and their derivatives by techniques such as inductively coupled plasma (ICP) or flame atomic absorption spectrometry (FAAS) analysis is essential for an accurate assessment of configurational entropy and proper classification. Furthermore, journal editors and reviewers should emphasize the importance of including these calculations in submitted works, helping to mitigate misinterpretations and maintain rigor in the field.

**Scheme 2:**
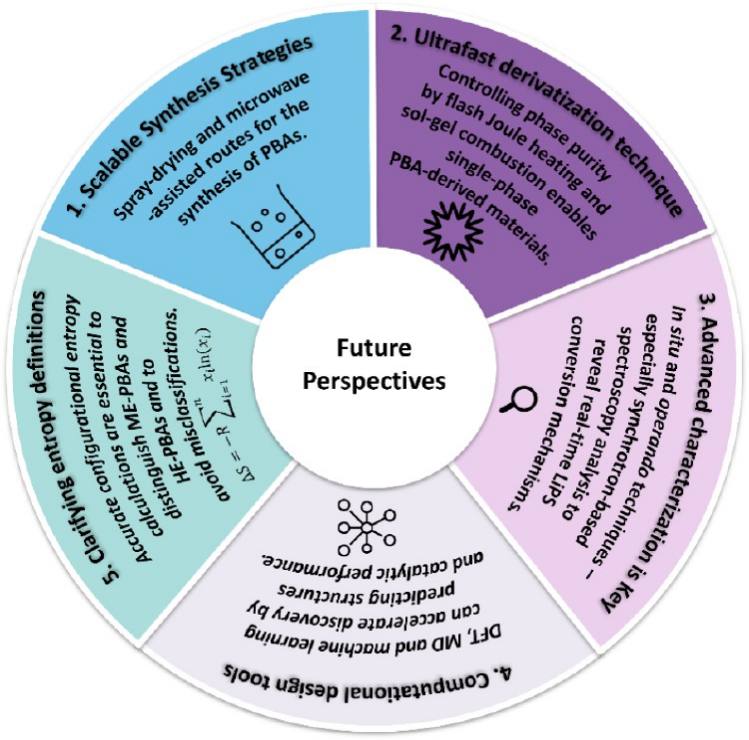
Schematic Representation Outlining Five Potential Research Pathways and Opportunities that Warrant Ongoing Exploration for the Development of Next-Generation Catalysts Based on PBAs and/or Their Derivatives

In summary, recent advancements in the application of low-, medium-, and high-entropy PBA-based catalytic materials and their derivatives for energy-related technologies highlight both promising progress and persistent challenges. While significant strides have been made, substantial efforts are still required to fully address the demands of our increasingly energy-dependent society.
